# The TRPM7 inhibitor carvacrol suppresses angiogenesis and vasculogenic mimicry in triple-negative breast cancer

**DOI:** 10.7150/ijbs.130027

**Published:** 2026-05-18

**Authors:** Tianci Tang, Na Zhao, Moqin Qiu, Luisa Müller, Michael D. Menger, Vladimir Chubanov, Thomas Gudermann, Gabriela Krasteva-Christ, Matthias W. Laschke, Yuan Gu

**Affiliations:** 1 Institute for Clinical and Experimental Surgery, Saarland University, PharmaScienceHub (PSH), 66421 Homburg, Germany.; 2 Institute of Anatomy and Cell Biology, Saarland University, 66421 Homburg, Germany.; 3 Walther-Straub Institute of Pharmacology and Toxicology, LMU Munich, 80336 Munich, Germany.; 4 Department of Respiratory Oncology, Guangxi Medical University Cancer Hospital, 530021 Nanning, China.

**Keywords:** carvacrol, angiogenesis, vasculogenic mimicry, TRPM7, mTOR, triple-negative breast cancer

## Abstract

Vasculogenic mimicry (VM) contributes significantly to tumor aggressiveness and resistance to anti-angiogenic therapies. Simultaneous inhibition of both angiogenesis and VM represents a promising strategy to improve therapeutic outcomes in aggressive cancers, such as triple-negative breast cancer (TNBC), which responds poorly to anti-angiogenic therapies. In this study, we identified carvacrol, a natural monoterpenoid phenol widely used as a food additive, as a dual inhibitor of angiogenesis and VM in TNBC. Carvacrol preferentially inhibited angiogenesis in endothelial cells (ECs) and VM in TNBC cells at concentrations that had minimal effects on TNBC cell proliferation. Mechanistically, carvacrol directly bound to the vanilloid-like (VL) site of transient receptor potential melastatin 7 (TRPM7), thereby inhibiting channel activity and attenuating Zn^2+^ influx. This triggered dephosphorylation of the mammalian target of rapamycin (mTOR) and subsequent proteasomal and lysosomal degradation of key receptor tyrosine kinases (RTKs), including vascular endothelial growth factor receptor 2 (VEGFR2), Tie2, fibroblast growth factor receptor 1 (FGFR1), and insulin-like growth factor 1 receptor (IGF1R) in ECs, as well as FGFR1 and IGF1R in TNBC cells. Genetic knockdown of TRPM7 recapitulated the anti-vascular effects and signaling alterations induced by carvacrol. *In vivo*, carvacrol effectively suppressed TNBC vascularization and growth in a mouse dorsal skinfold chamber model and an orthotopic xenograft model. Together, these findings suggest that carvacrol preferentially targets angiogenesis and VM in TNBC by suppressing the TRPM7/Zn^2+^/mTOR/RTKs axis, highlighting it as a promising therapeutic candidate for TNBC and potentially other tumors resistant to anti-angiogenic therapies, while positioning the TRPM7 channel as a novel anti-vascular target for TNBC treatment.

## Introduction

The rapid growth of malignant solid tumors requires an efficient blood vessel network to supply oxygen and nutrients and remove waste 1. Therefore, inhibition of tumor vascularization represents a promising therapeutic strategy for highly vascularized tumors, such as triple-negative breast cancer (TNBC). TNBC, lacking estrogen receptor, progesterone receptor, as well as human epidermal growth factor receptor 2 (HER2), is the most aggressive breast cancer subtype with very poor prognosis and limited treatment options 2.

Tumor vascularization primarily relies on angiogenesis, the development of new blood vessels from pre-existing ones 3. This is driven by pro-angiogenic factors within the tumor microenvironment, which bind to receptors on endothelial cells (ECs), stimulating their proliferation, migration, and organization into a new microvascular network 4. Receptor tyrosine kinases (RTKs), such as vascular endothelial growth factor receptor (VEGFR), Tie2, and fibroblast growth factor receptor (FGFR), play a dominant role in this process 5, 6. So far, approximately 20 anti-angiogenic agents targeting vascular endothelial growth factors (VEGFs) and RTKs, including monoclonal antibodies and small-molecule inhibitors, have been approved by the United States Food and Drug Administration (FDA) for the treatment of different types of cancer 7, 8. However, their clinical efficacy is significantly limited by the development of drug resistance. TNBC exemplifies this challenge, as it shows particularly poor responsiveness to anti-angiogenic therapies, precluding FDA approval of any such agents for this aggressive subtype.

One critical mechanism contributing to this resistance or unresponsiveness is vasculogenic mimicry (also called vascular mimicry; VM), a non-angiogenic mode of tumor vascularization 9. During VM, tumor cells acquire EC-like characteristics and functions, form tubular structures, and establish a blood vessel mimicry system independently of ECs 10. VM has been detected in a variety of tumor types, such as TNBC, and is closely associated with a high risk of tumor metastasis, poor clinical outcomes, and worse overall survival in cancer patients 10, 11. Notably, previous studies reported that the anti-angiogenic agent sunitinib promotes TNBC metastasis through VM, potentially explaining the limited efficacy of this class of drugs in TNBC clinical trials 12, 13. Accordingly, simultaneous inhibition of both angiogenesis and VM holds great promise for effectively disrupting blood supply and consequently hindering tumor growth, particularly in aggressive tumors that are unresponsive or resistant to anti-angiogenic therapies.

Phytochemicals serve as a valuable reservoir of potential candidates for anti-cancer drugs, given their low toxicity, easy accessibility, and diverse biological activities 14, 15. In fact, approximately 50% of all anti-cancer drugs currently on the market are derived from natural compounds 16. Carvacrol, a naturally occurring monoterpenoid phenol, is abundant in the essential oils of multiple aromatic herbs, such as oregano, thyme, and pepperwort 17. It is widely used as a fragrance ingredient in cosmetics and a safe flavoring additive in baked foods, chewing gum, and beverages 18. Previous studies have extensively reported its potent anti-cancer activity in different tumor types, which is primarily attributed to its cytotoxic and anti-proliferative effects 19. Nonetheless, the specific impact of carvacrol on tumor angiogenesis and VM, as well as its therapeutic potential in TNBC, remains to be elucidated.

Carvacrol was identified by Parnas *et al.* as an inhibitor of transient receptor potential melastatin 7 (TRPM7) channel activity in human embryonic kidney 293 (HEK293) cells overexpressing TRPM7 20. TRPM7 is a bifunctional protein comprising both ion channel and kinase domains, and its channel is highly permeable to divalent cations essential for cellular functions, such as Ca²⁺, Mg²⁺, and Zn²⁺ 21. It is expressed in multiple cancer types, where it regulates tumor cell proliferation, migration, and invasion 22. These findings raise the possibility that the anti-cancer effects of carvacrol may be mediated through the modulation of TRPM7. However, the role of TRPM7 in angiogenesis remains controversial, and its involvement in VM is largely unexplored. Furthermore, while TRPM7 and RTKs are recognized as independent contributors to tumor progression, their potential coordination as an integrated signaling axis in driving these specialized vascular processes in TNBC has yet to be established.

The present study seeks to address these knowledge gaps and evaluate the therapeutic potential of carvacrol in TNBC. Initially, we compared the effects of carvacrol on the viability of several types of primary ECs and TNBC cell lines. Subsequently, we examined the impact of carvacrol at non-cytotoxic concentrations (50, 100, and 200 µM) on key angiogenic activities of human umbilical vein endothelial cells (HUVECs), including their proliferation, migration, tube formation, and spheroid sprouting. These *in vitro* angiogenesis assays were complemented by an *ex vivo* aortic ring assay and an *in vivo* Matrigel plug assay. In parallel, we investigated the effects of carvacrol at the same concentrations on TNBC cell proliferation, migration, and VM using MDA-MB-231, HCC1937, and 4T1 cell lines. In addition, we clarified the precise molecular mechanisms underlying the anti-vascular activity of carvacrol. Finally, we evaluated the effects of carvacrol on angiogenesis, VM, and growth of TNBC in a mouse dorsal skinfold chamber model and an orthotopic xenograft model.

## Materials and methods

### Study design

The sample size for each experiment was determined based on previously published studies. For *in vitro* assays, a minimum of 3 independent experiments were performed, each consisting of at least 3 biological replicates (i.e., independent cell cultures). For mouse experiments, group sizes (n = 7-8) were determined based on previous publications, pilot observations, and the 3Rs principle to minimize animal usage while maintaining scientific reliability. To validate the adequacy of group sizes, a *post-hoc* power analysis was performed using G*Power 3.1.9.7 (Heinrich Heine University Düsseldorf) based on the primary endpoints (microvessel density or tumor size at the final time point). This analysis revealed a large experimental effect size (Cohen's d > 1.5) and a high statistical power (> 80%; α = 0.05) across all mouse models used in this study, confirming that the study was sufficiently powered to detect biologically meaningful differences. Random allocation was applied for group assignments in both the dorsal skinfold chamber model and the orthotopic xenograft model. Data analysis was conducted by investigators blinded to the group assignments. No samples or animals were excluded from the analysis. Exact n values for each experiment are provided in the corresponding figure legends.

### Chemicals

Chemicals used in this study included carvacrol (purity: 98%; 282197; Sigma-Aldrich; Merck KGaA, Darmstadt, Germany), chloroquine diphosphate salt (CQ; C6628; Sigma-Aldrich), MG132 (133407-82-6; Santa Cruz Biotechnology, Heidelberg, Germany), A-967079 (HY-108463; MedChemExpress, NJ, USA), 74a (HY-131868; MedChemExpress), AMTB hydrate (SML0103; Sigma-Aldrich), naltriben methanesulfonate hydrate (N156; Sigma-Aldrich), CaCl₂ (C-34006; PromoCell, Heidelberg, Germany), ZnSO₄ (83265; Sigma-Aldrich), MgSO₄ (M3409; Sigma-Aldrich), and MHY1485 (HY-B0795; MedChemExpress).

### Cell culture

HUVECs (passages 5-8; PromoCell) were cultured in Endothelial Cell Basal Medium (EBM; PromoCell) supplemented with SupplementMix (PromoCell). Human dermal microvascular endothelial cells (HDMECs; passages 5-8; PromoCell) were maintained in EBM-MV (PromoCell) supplemented with SupplementMix (PromoCell). Murine TNBC 4T1 cells (RRID:CVCL_A4BM; ATCC, Wesel, Germany) and human TNBC HCC1937 cells (ATCC) were cultured in RPMI 1640 medium (PAN-Biotech GmbH, Aidenbach, Germany) supplemented with 10% fetal calf serum (FCS; PAN-Biotech), 100 U/mL penicillin (PAN-Biotech), and 0.1 mg/mL streptomycin (PAN-Biotech). Human TNBC MDA-MB-231 cells expressing luciferase (MDA-MB-231-Luc; RRID:CVCL_C9CE; GeneCopoeia, Heidelberg, Germany) and HEK293T cells (RRID:CVCL_0063; ATCC) were cultured in Dulbecco's modified Eagle's medium (DMEM; PAN-Biotech) containing 10% FCS, 100 U/mL penicillin, and 0.1 mg/mL streptomycin. All cell lines were cultured in an incubator at 37 °C under a humidified atmosphere with 5% CO_2_.

### Cell transfection

For TRPM7 knockdown, HUVECs and MDA-MB-231-Luc cells were transfected with 150 nM small interfering RNAs (siRNAs) against TRPM7 (si-TRPM7; ON-TARGETplus siRNA SMARTpool, Dharmacon, Colorado, USA) using HiPerFect reagent (Qiagen, Hilden, Germany) according to the manufacturer's instructions. Negative control siRNA (si-NC; Qiagen) served as the control. After 48 h, cells were trypsinized, counted, and equal numbers of cells from each group were used for subsequent assays. For TRPM7 overexpression, HEK293T cells seeded in 35-mm dishes were transfected with 2 µg of plasmid DNA encoding wild-type *Trpm7* or point mutants (A981L and W1111A) cloned into the pIRES2-EGFP expression vector using 6 µL of FuGENE^®^ HD transfection reagent (Promega, Walldorf, Germany) according to the manufacturer's instructions. The generation of expression vectors was described previously 23, 24. After 48 h, EGFP^+^ cells were selected for whole-cell patch-clamp recordings.

### Lactate dehydrogenase (LDH) assay

An LDH assay was performed to assess the cytotoxicity of carvacrol according to the manufacturer's protocol (Roche Diagnostics, Mannheim, Germany). Briefly, HUVECs (4 × 10³ cells/well) were seeded in 96-well plates and treated with a serial dilution of carvacrol for 24 h. Then, 100 μL of reaction mixture containing catalyst and dye was added to each well. After a 10-min incubation at room temperature, 50 µL of stop solution was added to terminate the reaction. The activity of released LDH was determined by measuring the absorbance at 492 nm with 620 nm as reference using a microplate photometer (PHOmo; anthos Mikrosysteme GmbH, Krefeld, Germany). Cytotoxicity was calculated using the formula: Cytotoxicity (%) = (OD_sample_-OD_0µM_) / (OD_high control_-OD_0µM_) × 100. The high control, representing total cell death, was generated by treating cells with 5 µL of lysis solution.

### Bromodeoxyuridine (BrdU) incorporation assay

A BrdU incorporation assay was conducted to analyze cell proliferation. HUVECs (2.5 × 10^5^ cells/well) or MDA-MB-231-Luc cells (1.5 × 10^5^ cells/well) were cultured in 6-well plates and then exposed to different concentrations of carvacrol. After 6 h of treatment with carvacrol, BrdU reagent was added to the culture medium at a final concentration of 10 µM. Following an 18-h incubation with both carvacrol and BrdU, the cells were fixed with 70% ethanol on ice for 30 min and denatured in 2 M hydrochloric acid containing 0.5% Triton X-100 for another 30 min at room temperature. They were then incubated with a fluorescein isothiocyanate (FITC)-labeled anti-BrdU antibody (1:30; 11-5071-42; RRID:AB_11042627; Thermo Fisher Scientific, Karlsruhe, Germany) for 1 h at room temperature. The percentage of FITC^+^ proliferating cells was measured using a FACSLyric flow cytometer (BD Biosciences, Heidelberg, Germany).

### Transwell migration assay

A Transwell migration assay was performed to analyze cell motility using 24-well Transwell plates with 8 μm pore polyester membrane inserts (Corning, Merck KGaA). Briefly, HUVECs or MDA-MB-231 cells grown to 70-80% confluence in 100-mm dishes were treated with different concentrations of carvacrol for 24 h. Thereafter, 500 μL of cell suspension containing 5 × 10^4^ HUVECs or 3.5 × 10^4^ MDA-MB-231 cells in EBM or DMEM without supplements was added to each insert, while 750 μL of EBM or DMEM containing 1% FCS was added to the lower chamber. After 5 h of incubation, non-migrated cells were gently removed from the upper side of the insert membrane with a cotton swab. Migrated cells were then stained with Diff-Quick (LT-SYS Diagnostika, Berlin, Germany). At least 20 random non-overlapping fields were imaged at a 200-fold magnification with a phase-contrast microscope (BZ-8000; Keyence, Osaka, Japan). The number of migrated cells in each field was quantified using ImageJ software (U.S. National Institutes of Health, Bethesda, Maryland, USA).

### Tube formation assay

A tube formation assay served to assess the capability of ECs or tumor cells to form capillary-like structures. For this purpose, 50 μL of Matrigel (Corning) was added to each well of 96-well plates on ice and then incubated at 37 °C for 15 min to solidify the gel. Then, 100 μL of cell suspension containing 1.7 × 10^4^ HUVECs or 6 × 10^4^ MDA-MB-231-Luc cells with different concentrations of carvacrol was added onto the gel. After 18 h of incubation, images of the cells were acquired at 20-fold magnification using a phase-contrast microscope (BZ-8000). The number of formed tube meshes was quantified using ImageJ software with the Angiogenesis Analyzer plugin.

### Spheroid sprouting assay

A spheroid sprouting assay was performed following a previously established protocol with slight modifications 25. In brief, 500 HUVECs in 50 μL of growth medium containing 0.24% methylcellulose (Thermo Fisher Scientific) were seeded into each well of 96-well round-bottom, non-adherent plates to generate spheroids (Greiner Bio-One, Frickenhausen, Germany). After 24 h of incubation, the spheroids were collected and resuspended in 300 μL of polymerization solution, which was prepared by mixing collagen solution with EBM containing 20% FCS and 0.5% methylcellulose at a ratio of 1:1. The collagen solution was composed of rat acidic collagen extract (4 mg/mL; Advanced Biomatrix, Carlsbad, USA), H_2_O, 10 × Medium 199 (Sigma-Aldrich), and 0.2 M sodium hydroxide at a 4:4:1:1 ratio. The spheroids (~50/well) were then rapidly transferred into a pre-warmed 24-well plate and incubated at 37°C for 45 min, after which 500 μL of growth medium containing different concentrations of carvacrol was added to each well. After 24 h of treatment, images of the spheroids were acquired using a phase-contrast microscope (DFC450C; Leica Microsystems, Wetzlar, Germany). The cumulative sprout length of each spheroid was quantified using the LAS V4.8 software (Leica Microsystems).

### Aortic ring assay

An aortic ring assay was performed to investigate the effects of carvacrol on aortic sprouting. First, 96-well plates were coated with Matrigel (40 µL/well; Corning) and incubated for 10 min at 37 °C to allow Matrigel polymerization. Thoracic aortas were isolated from 8-week-old male BALB/c mice (RRID:IMSR_RJ:BALB-CANNRJ; Janvier-Labs, Le Genest, France) under sterile conditions and cut into approximately 1 mm-long rings. Subsequently, another 40 µL of Matrigel was added to each well, followed by placing a single aortic ring in the center. After the Matrigel solidified, 100 µL of DMEM supplemented with 10% FCS and different concentrations of carvacrol was gently added onto the gel. After 3 days, carvacrol was refreshed along with medium change. On day 6 after treatment, the aortic rings were imaged with a phase-contrast microscope (BZ-8000). The sprouting area of each aortic ring was measured using image analysis software (Keyence).

### Western blotting

Total protein lysates were prepared using ice-cold radioimmunoprecipitation assay (RIPA) lysis buffer (Thermo Fisher Scientific) supplemented with protease and phosphatase inhibitors (Sigma-Aldrich). Protein quantification was performed using the bicinchoninic acid (BCA) protein assay kit (Thermo Fisher Scientific). Subsequently, protein samples were diluted in 6× Laemmli buffer (Sigma-Aldrich), boiled for 5 min, and separated by 8% or 12% sodium dodecyl sulfate-polyacrylamide gel electrophoresis. The separated proteins were then transferred onto polyvinylidene fluoride membranes (Bio-Rad, Munich, Germany). These membranes were further blocked with 5% bovine serum albumin at room temperature for 1 h and then probed overnight at 4 °C with primary antibodies, including a rabbit anti-VEGFR1 antibody (1:100; ab32152; RRID:AB_778798; Abcam, Cambridge, UK), a rabbit anti-VEGFR2 antibody (1:250; 9698; RRID:AB_11178792; Cell Signaling Technology, Frankfurt, Germany), a rabbit anti-Tie2 antibody (1:250; 7403; RRID:AB_10949315; Cell Signaling Technology), a rabbit anti-FGFR1 antibody (1:100; 9740; RRID:AB_11178519; Cell Signaling Technology), a rabbit anti-insulin-like growth factor 1 receptor (IGF1R) antibody (1:100; 9750T; RRID:AB_10950969; Cell Signaling Technology), a rabbit anti-epidermal growth factor receptor (EGFR) antibody (1:250; 4267T; RRID:AB_2246311; Cell Signaling Technology), a rabbit anti-phosphorylated mammalian target of rapamycin (p-mTOR) antibody (1:250; 5536; RRID:AB_10691552; Cell Signaling Technology), a rabbit anti-mTOR antibody (1:250; 2983; RRID:AB_2105622; Cell Signaling Technology), a rabbit anti-p62 antibody (1:250; 5114T; RRID:AB_10624872; Cell Signaling Technology), a rabbit anti-LC3B antibody (1:250; 3868T; RRID:AB_2137707; Cell Signaling Technology), a rabbit anti-TRPM7 antibody (1:100; 55251-1-AP; RRID:AB_2935541; Proteintech), and a mouse anti-β-actin antibody (1:250; A5441; RRID:AB_476744; Sigma-Aldrich). This was followed by incubation with an anti-rabbit (1:1000; HAF008; RRID:AB_357235; R&D Systems, Wiesbaden, Germany) or anti-mouse (1:1000; HAF007; RRID:AB_357234; R&D Systems) secondary antibody conjugated to horseradish peroxidase for 1 h at room temperature. Signal development was achieved using the enhanced chemiluminescence kit (Bio-Rad) and images were captured with a ChemoCam Imager (Intas, Göttingen, Germany).

### Quantitative real-time polymerase chain reaction (qRT-PCR)

After exposure to 0.1% DMSO (vehicle) or 200 µM carvacrol for 4 h, HUVECs were collected for RNA extraction using the RNeasy Mini kit (Qiagen) according to the manufacturer's protocol. Afterwards, cDNA was synthesized using the QuantiTect Reverse Transcription Kit (Qiagen). qRT-PCR analysis was carried out using the QuantiTect SYBR Green PCR kit (Qiagen) on a CFX96 Real-Time PCR System (Bio-Rad). The messenger RNA (mRNA) levels of target genes were normalized to glyceraldehyde-3-phosphate dehydrogenase (GAPDH) expression and quantified using the 2^-ΔΔCt^ method. The primer sequences were as follows: 5'-GGCCCAATAATCAGAGTGGCA-3' (forward) and 5'-CCAGTGTCATTTCCGATCACTTT-3' (reverse) for human VEGFR2; 5'-TTAGCCAGCTTAGTTCTCTGTGG-3' (forward) and 5'-AGCATCAGATACAAGAGGTAGGG-3' (reverse) for human Tie2; 5'-GGCTACAAGGTCCGTTATGCC-3' (forward) and 5'-GATGCTGCCGTACTCATTCTC-3' (reverse) for human FGFR1; 5'-TCGACATCCGCAACGACTATC-3' (forward) and 5'-CCAGGGCGTAGTTGTAGAAGAG-3' (reverse) for human IGF1R; 5'-ATGGGTGTGAACCATGAGAAGTA-3' (forward) and 5'-GGCAGTGATGGCATGGAC-3' (reverse) for human GAPDH.

### Water-soluble tetrazolium (WST)-1 assay

A WST-1 assay was performed to analyze cell viability. For this, cells of different types were seeded in 96-well plates at a density of 2-3 × 10^3^ cells/well. After overnight incubation, they were treated with a serial dilution of different chemicals for 24 h or 48 h. Then, 10 μL of WST-1 reagent (Roche Diagnostics) was added to each well, followed by incubation at 37 °C for 30 min. Thereafter, the absorbance of each well was measured at 450 nm with 620 nm as reference in a microplate photometer (PHOmo). Cell viability was calculated using the formula: Viability (%) = (OD_sample_-OD_medium_) / (OD_0µM_-OD_medium_) × 100. The medium control was only medium without cells.

### Whole-cell patch-clamp measurements

HUVECs or EGFP^+^ HEK293T cells expressing TRPM7 were seeded onto 25-mm glass coverslips and mounted in a recording chamber, continuously perfused with Mg^2+^-free Tyrode solution containing 140 mM NaCl, 5 mM KCl, 10 mM HEPES, 10 mM glucose, and 2 mM CaCl_2_ (pH~7.4), as described previously 26. Cells were patched using a Nikon microscope (TE2000e, Tokyo, Japan) with a 40-fold water-immersion objective and filter sets for GFP excitation provided by an LED illumination lamp (CoolLED, Andover, UK). Images were acquired using an ORCA-spark Digital CMOS camera (Hamamatsu Photonics, Hamamatsu, Japan). Whole-cell membrane currents were recorded using an EPC 10 USB amplifier (HEKA Elektronik GmbH, Lambrecht, Germany), low-pass filtered at 3 kHz, and data acquisition was controlled by the PatchMaster software (HEKA Elektronik GmbH). Patch pipettes (resistance: 3-5 MΩ) were pulled from borosilicate capillaries (outside diameter: 1.5 mm; inside diameter: 1.05 mm; Science Products, Hofheim am Taunus, Germany) and polished using a DMZ universal electrode puller (Zeitz-Instrumente, Planegg, Germany). The pipettes were filled with an intracellular solution containing 120 mM CsCl, 8 mM NaCl, 10 mM HEPES, 1 mM EGTA, and 0.3 mM CaCl_2_ (pH~7.2), yielding a free Ca²⁺ concentration of 109 nM, calculated using WebMaxC. TRPM7 currents were elicited by a ramp voltage from -100 mV to +100 mV over 100 ms with a holding potential of 0 mV at a frequency of every 5 s. Currents measured at +80 mV were analyzed for all experiments. Further data analysis was performed using Igor Pro 8 (WaveMetrics, Inc., USA) and GraphPad Prism 10.4.1. All recordings were performed at room temperature.

### Blind molecular docking

The crystal structure of C-terminally truncated mouse TRPM7 (PDB ID: 8W2L) was retrieved from the Protein Data Bank (https://www.rcsb.org) and prepared using UCSF ChimeraX (version 1.8) by removing water molecules and heteroatoms. Polar hydrogen atoms were added and Gasteiger charges were assigned using AutoDockTools (version 1.5.7), and the prepared receptor was saved in PDBQT format. Three-dimensional (3D) structures of carvacrol, CCT128930, and NS8593 were obtained from PubChem (https://pubchem.ncbi.nlm.nih.gov). Ligand preparation was conducted using Avogadro 2 (version 1.99.0) for hydrogen addition and geometry optimization, followed by the definition of rotatable bonds and conversion to PDBQT format using AutoDockTools. Blind docking simulations were performed using AutoDock 4.2 with a Lamarckian genetic algorithm. A grid box encompassing the entire TRPM7 structure was configured with a grid spacing of 1.0 Å. Each ligand underwent 100 independent docking runs with standard parameters (population size: 150; energy evaluations: 2,500,000). Docking conformations were clustered based on root-mean-square deviation and the lowest-energy conformation was selected for analysis. Binding free energy (ΔG, kcal/mol) was calculated, with more negative values indicating stronger binding affinity. Protein-ligand interactions were analyzed using BIOVIA Discovery Studio Visualizer 2025 and visualized with UCSF ChimeraX.

### Animal experiments

The mice were housed in a conventional animal facility (Institute for Clinical and Experimental Surgery, Saarland University, Homburg, Germany) under a 12-h light/12-h dark cycle and were given free access to pellet food (Altromin, Lage, Germany) and water.

A Matrigel plug assay was performed to assess the *in vivo* effects of carvacrol on angiogenesis. For this purpose, 250 μL of growth factor-reduced Matrigel (Corning) supplemented with 1 µg/mL VEGF (R&D Systems), 1 µg/mL basic fibroblast growth factor (bFGF; R&D Systems), 50 IU/mL heparin (B. Braun, Melsungen, Germany), 0.1% DMSO (vehicle) or 200 µM carvacrol was injected subcutaneously into the flanks of 3-month-old male BALB/c mice (25-30 g; Janvier-Labs; n = 8 per group). Before the injection, the mice were anesthetized with isoflurane (5% induction and 2% maintenance). On day 7 after injection, the Matrigel plugs were dissected and fixed in 4% formalin for immunohistochemical analyses.

A dorsal skinfold chamber model was used to evaluate the impact of carvacrol on mouse TNBC vascularization and growth, following a previously detailed protocol with modifications 27. Tumor spheroids were generated by seeding 4T1 cells (5 × 10^4^ cells/well) into 96-well plates coated with 1% agarose and incubating them for 3 days. One day after cell seeding, dorsal skinfold chambers were implanted in 3-month-old female BALB/c mice (22-25 g; Janvier-Labs). Prior to the operation, the mice were anesthetized with an intraperitoneal injection of ketamine (Ketabel^®^; 90 mg/kg body weight; Serumwerke Bernburg AG, Bernburg, Germany) and xylazine (Rompun^®^; 12 mg/kg body weight; Bayer, Leverkusen, Germany). For post-operative analgesia, they received a subcutaneous injection of carprofen (Rimadyl^®^; 10 mg/kg body weight; Cp-Pharma, Burgdorf, Germany). After another 2 days, one 4T1 spheroid was transplanted into each chamber. Subsequently, the mice were randomly divided into two groups (n = 7 per group) and received 50 mg/kg body weight carvacrol or a vehicle solution (a mixture containing 5 µL of DMSO and 45 µL of 20% SBE-β-CD in saline) via intraperitoneal injection once daily for 14 days. This dosage of carvacrol was selected based on previously reported in *vivo* efficacy in different murine disease models 28-31 and toxicological data demonstrating that 50 mg/kg remains within a tolerable, sub-lethal range below the median lethal dose (LD_50_) of 73.3 mg/kg for this administration route 32. Intravital fluorescence microscopy was performed on days 0, 3, 6, 10, and 14 following spheroid transplantation using a Zeiss Axiotech microscope (Zeiss, Oberkochen, Germany) equipped with a charge-coupled device video camera (FK6990; Pieper, Schwerte, Germany). To visualize the functional microvasculature, mice received an intravenous injection of 5% FITC-dextran (MW 150,000; 0.1 mL). The following parameters were analyzed off-line using the CapImage analysis system (Zeintl, Heidelberg, Germany): Functional microvessel density was calculated as the total length of red blood cell (RBC)-perfused vessels divided by the area of observation. Tumor size was determined as the total surface area of the tumors. Microvessel diameter (D) was measured perpendicularly to the vessel path. Centerline RBC velocity was assessed using the line-shift method, as previously described 33, 34. This method is based on the measurement of the shift of an individual intravascular gray-level pattern along the vessel centerline over a fixed time interval. Volumetric blood flow was calculated based on the measured vessel diameter (D) and centerline velocity (V) using the formula: Qv = π × (D/2)² × V/1.3, where 1.3 represents an empirical correction factor for converting centerline velocity to mean blood flow velocity in tumor microvessels 35, 36. On day 14, the tumor tissues were excised for further histological and immunohistochemical analyses.

An orthotopic xenograft model was employed to investigate the effects of carvacrol on the vascularization and growth of human TNBC. Briefly, 5 × 10^6^ MDA-MB-231-Luc cells suspended in 50 μL of PBS were injected into the left fourth mammary fat pad of 6-week-old female NOD-SCID mice (22-25 g; RRID:IMSR_RJ:NOD-SCID; Janvier-Labs). After 3 days, when the tumor became palpable, the mice were randomly assigned to two groups (n = 7 per group) and treated with 50 mg/kg body weight carvacrol or a vehicle solution (a mixture containing 5 µL of DMSO and 45 µL of 20% SBE-β-CD in saline) by intraperitoneal injection once daily until 6 weeks after tumor inoculation. During this period, two perpendicular diameters of the tumors were measured weekly with a digital caliper. Tumor volume (V) was calculated using the following formula: V = 0.5 × length × width^2^ 37. Tumor growth was also analyzed weekly by bioluminescence imaging using an IVIS Spectrum imaging system (PerkinElmer, MA, USA). To achieve this, the mice bearing tumors were administered 150 mg/kg body weight D-luciferin (122799; PerkinElmer) via intraperitoneal injection and then anesthetized with isoflurane (5% induction and 2% maintenance). Bioluminescent images were acquired 17 min after D-luciferin injection and analyzed using the Living Image software (PerkinElmer) to quantify the total flux of the bioluminescent signal within the tumor regions. On day 42 after tumor inoculation, the tumor tissues were harvested, weighed, photographed, and fixed in 4% formalin for further immunohistochemical analyses.

### Zn^2+^ quantification assay

Intratumoral Zn^2+^ levels were quantified using a colorimetric Zinc Assay Kit (ab102507; Abcam) according to the manufacturer's instructions. Briefly, tumor tissues were homogenized in a cold HEPES/KCl buffer (25 mM HEPES, 100 mM KCl; pH 7.0) at a ratio of 100 mg tissue per mL buffer. Then, 100 μL of tumor lysate was mixed with 100 μL of 7% trichloroacetic acid to precipitate proteins and release protein-bound Zn^2+^. The samples were then centrifuged at 16,000 × g for 5 min at 4 °C, and a 50 μL aliquot of the supernatant was transferred to a 96-well plate. Subsequently, 200 μL of zinc reaction mixture (Zinc Reagent 1 and Zinc Reagent 2 mixed at a 4:1 ratio) was added to each well, followed by incubation for 10 min at room temperature. The absorbance was measured at 560 nm using a microplate reader (Infinite 200; Tecan, Männedorf, Switzerland). Zn^2+^ concentrations were determined using a standard curve and normalized to the tumor tissue wet weight (expressed as μg/g tissue).

### Histology and immunohistochemistry

Matrigel plugs and tumor tissues, fixed in formalin, were sequentially dehydrated in ethanol and embedded in paraffin. Then, 3-µm-thick tissue slices were serially cut and mounted onto slides.

To detect microvessels in Matrigel plugs, the sections were incubated with a rabbit anti-mouse CD31 antibody (1:100; ab182981; RRID:AB_2920881; Abcam) overnight at 4℃. Subsequently, they were incubated with a goat anti-rabbit Alexa Fluor 555-labeled secondary antibody (1:100; A27039; RRID:AB_2536100; Thermo Fisher Scientific) and Hoechst 33342. The entire area of each sample was imaged under a BX-60 microscope (Olympus, Tokyo, Japan) at a 400-fold magnification. The number of CD31^+^ microvessels in each field was quantified using ImageJ software.

For tumor size analysis, the sections with the largest tumor area were stained with hematoxylin and eosin (HE). After this, images were taken using a phase-contrast microscope (BZ-8000) and analyzed using image analysis software (Keyence) to quantify the tumor area.

To assess tumor cell proliferation and apoptosis, sections were sequentially incubated with a rabbit anti-mouse Ki67 antibody (1:400; 12202; RRID:AB_2620142; Cell Signaling Technology) or a rabbit anti-mouse cleaved caspase-3 antibody (1:100; 9661; RRID:AB_2341188; Cell Signaling Technology), a biotinylated goat anti-rabbit secondary antibody (1:100; ab64256; RRID:AB_2661852; Abcam), peroxidase-conjugated streptavidin (ready-to-use; Abcam), and 3-amino-9-ethylcarbazole substrate (Abcam). Finally, they were counterstained with Mayer's hemalum solution (Merck KGaA). The entire area of each sample was imaged under a BX-60 microscope at a 400-fold magnification. The percentage of Ki67^+^ or cleaved caspase-3^+^ tumor cells was quantified using ImageJ software.

To evaluate angiogenesis and VM in TNBC, CD31 and periodic acid-Schiff (PAS) double staining was carried out. Briefly, the tumor sections were sequentially stained with a rabbit anti-mouse CD31 antibody (1:100; ab182981; RRID:AB_2920881; Abcam), a biotinylated goat anti-rabbit secondary antibody (1:100; ab64256; RRID:AB_2661852; Abcam), peroxidase-conjugated streptavidin (Abcam), and 3-amino-9-ethylcarbazole substrate (Abcam). Subsequently, they were exposed to periodic acid and Schiff reagent (Sigma-Aldrich), followed by counterstaining with Mayer's hemalum solution (Merck KGaA). The entire area of each sample was imaged under a BX-60 microscope at a 400-fold magnification. The percentage of CD31^+^ PAS^+^ EC-lined vessels and CD31^-^ PAS^+^ VM structures was quantified using ImageJ software.

### Statistics

Statistical analysis was performed using GraphPad Prism 10.4.1. Differences between two groups were analyzed using an unpaired two-tailed t-test, whereas differences among more than two groups were analyzed using one-way ANOVA followed by Tukey's multiple comparisons test. All data were expressed as means ± standard error of the mean (SEM). A *P*-value < 0.05 was considered significant (*P < 0.05, **P < 0.01, ***P < 0.001).

## Results

### Carvacrol preferentially reduces EC viability compared to TNBC cells

**Figure 1:**
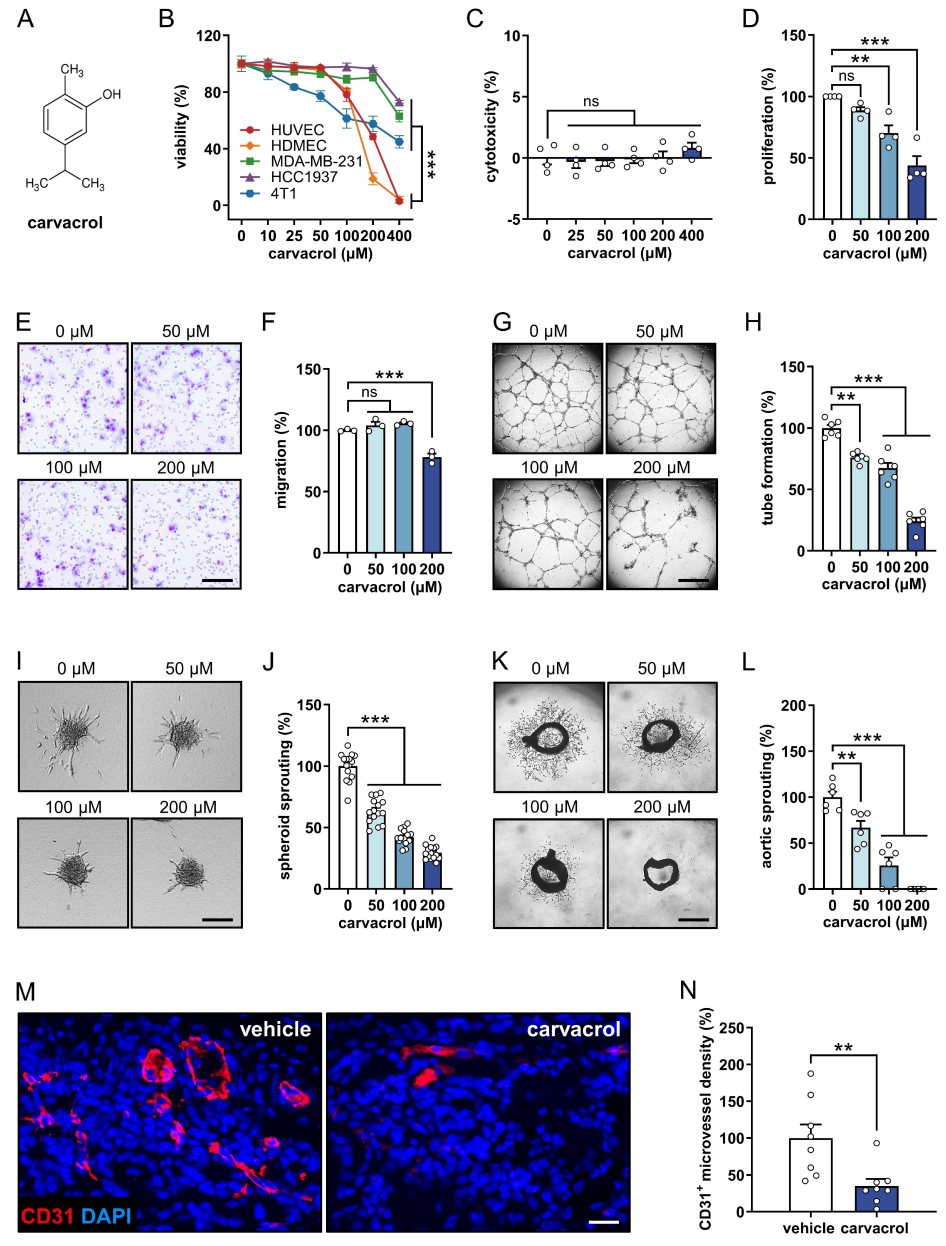
Carvacrol inhibits angiogenesis *in vitro*, *ex vivo*, and *in vivo*. **A:** Chemical structure of carvacrol. **B:** Viability (% of 0 µM) of HUVECs, HDMECs, MDA-MB-231, HCC1937, and 4T1 cells that were treated for 48 h with a serial dilution of carvacrol, as assessed by WST-1 assay (n = 4-5). **C:** Cytotoxicity (% of total cell death) of carvacrol against HUVECs, as assessed by LDH assay (n = 4). The cells were treated with a serial dilution of carvacrol for 24 h. **D:** Proliferation (% of 0 µM) of HUVECs that were treated for 24 h with 0, 50, 100, and 200 µM carvacrol, as assessed by BrdU incorporation assay (n = 4 independent experiments). **E:** Representative images of migrated HUVECs. The cells were treated with 0, 50, 100, and 200 µM carvacrol for 24 h prior to the assay. Scale bar: 70 µm. **F:** Migration (% of 0 µM) of treated HUVECs depicted in (E), as assessed by Transwell migration assay (n = 3). **G:** Representative images of tube-forming HUVECs that were treated for 18 h with 0, 50, 100, and 200 µM carvacrol. Scale bar: 720 µm. **H:** Tube formation (% of 0 µM) of treated HUVECs depicted in (G), as assessed by tube formation assay (n = 6). **I:** Representative images of HUVEC spheroids that were treated for 24 h with 0, 50, 100, and 200 µM carvacrol. Scale bar: 85 µm. **J:** Sprouting (% of 0 µM) of treated HUVEC spheroids depicted in (I), as assessed by spheroid sprouting assay (n = 15). **K:** Representative images of mouse aortic rings after 6-day treatment with 0, 50, 100, and 200 µM carvacrol. Scale bar: 1 mm. **L:** Sprouting (% of 0 µM) of treated aortic rings depicted in (K), as assessed by aortic ring assay (n = 6). **M:** Representative images of Matrigel plugs containing 0.1% DMSO (vehicle) or 200 µM carvacrol. The sections were stained with an anti-CD31 antibody (red) and Hoechst 33342 (blue) to visualize ECs and cell nuclei, respectively. Scale bar: 30 µm. **N:** CD31^+^ microvessel density (% of vehicle) in Matrigel plugs depicted in (M), as assessed by immunohistochemistry (n = 8). Data are presented as means ± SEM. **P < 0.01, ***P < 0.001; ns, not significant.

In a first set of experiments, we assessed the effects of carvacrol (chemical structure shown in Fig. 1A) on the viability of ECs (HUVECs and HDMECs) and TNBC cell lines (MDA-MB-231, HCC1937, and 4T1). WST-1 assays revealed that treatment with 400 µM carvacrol for 48 h preferentially and significantly reduces the viability of both tested EC types when compared to the TNBC cell lines (Fig. 1B).

### Carvacrol inhibits the angiogenic activity of ECs *in vitro*, *ex vivo*, and *in vivo*

To determine non-cytotoxic concentrations of carvacrol in HUVECs, we performed LDH assays. Our data showed that concentrations up to 400 µM of carvacrol exhibit no cytotoxicity against HUVECs after 24 h of treatment (Fig. 1C). Based on this finding, we selected concentrations of 50, 100, and 200 µM carvacrol for further angiogenesis assays.

BrdU incorporation assays revealed that 100 and 200 µM carvacrol significantly inhibit the proliferation of HUVECs (Fig. 1D). Treatment with 200 μM carvacrol also caused a 22% reduction in the number of migrated HUVECs (Fig. 1E, F). Moreover, this compound effectively inhibited EC tube formation (Fig. 1G, H) and spheroid sprouting (Fig. 1I, J) in a dose-dependent manner. Additional *ex vivo* aortic ring assays demonstrated that carvacrol dose-dependently suppresses the sprout outgrowth from aortic rings, with 200 µM completely blocking this process (Fig. 1K, L). Finally, we investigated the *in vivo* effects of carvacrol on angiogenesis using a Matrigel plug assay. This assay showed that Matrigel plugs containing 200 µM carvacrol exhibit a 65% reduction in the density of CD31^+^ microvessels when compared to controls (Fig. 1M, N).

### Degradation of VEGFR2, Tie2, FGFR1, and IGF1R contributes to the anti-angiogenic effect of carvacrol

**Figure 2:**
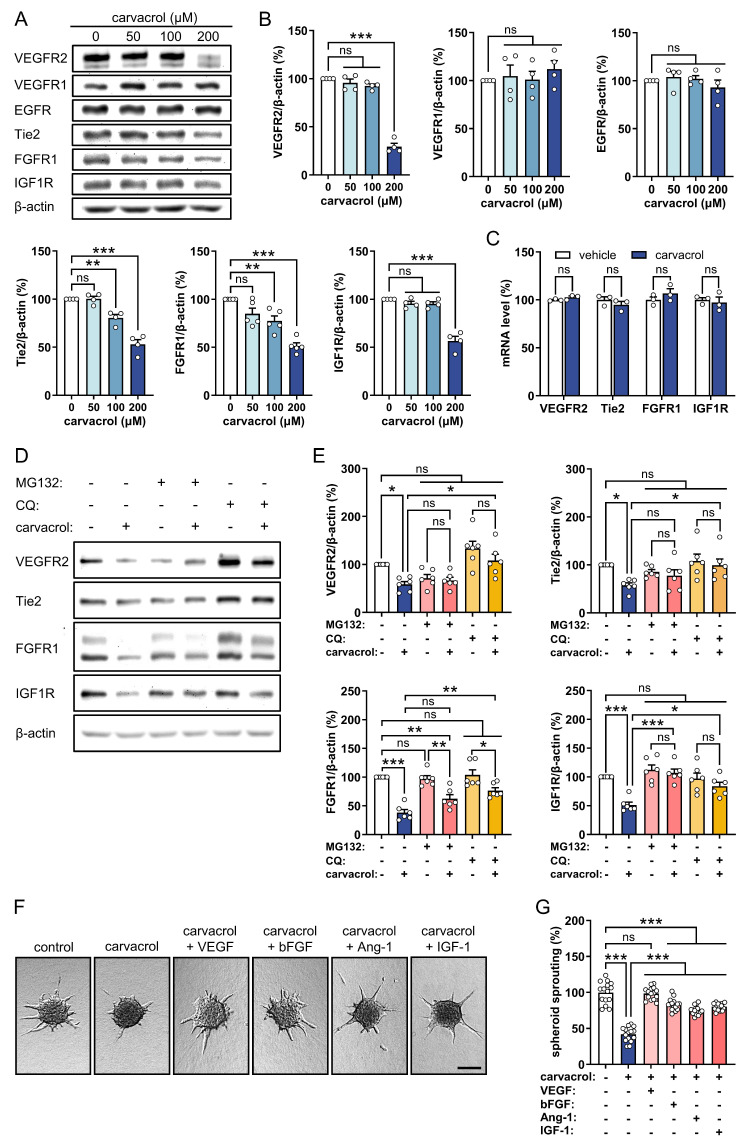
Carvacrol inhibits angiogenesis through promoting the degradation of VEGFR2, Tie2, FGFR1, and IGF1R in ECs. **A:** Representative Western blots showing VEGFR2, VEGFR1, EGFR, Tie2, FGFR1, IGF1R, and β-actin expression in HUVECs that were treated for 4 h with 0, 50, 100, and 200 µM carvacrol. **B:** Expression levels (% of 0 µM) of VEGFR2, VEGFR1, EGFR, Tie2, FGFR1, and IGF1R normalized to β-actin in treated HUVECs depicted in (A), as assessed by Western blotting (n = 4-5 independent experiments). **C:** mRNA levels (% of vehicle) of VEGFR2, Tie2, FGFR1, and IGF1R in HUVECs that were treated with 0.1% DMSO (vehicle) or 200 µM carvacrol for 4 h, as assessed by qRT-PCR (n = 3). **D:** Representative Western blots showing VEGFR2, Tie2, FGFR1, IGF1R, and β-actin expression in HUVECs that were pre-treated without or with 10 µM MG132 (proteasome inhibitor) or 200 µM CQ (lysosome inhibitor) for 2 h and then exposed to 0.1% DMSO (vehicle) or 200 µM carvacrol for another 4 h. **E:** Expression levels (% of control) of VEGFR2, Tie2, FGFR1, and IGF1R normalized to β-actin in treated HUVECs depicted in (D), as assessed by Western blotting (n = 6 independent experiments). **F:** Representative images of HUVEC spheroids that were treated for 24 h with 0.1% DMSO (vehicle) or 200 µM carvacrol in the absence or presence of 50 ng/mL VEGF, 100 ng/mL bFGF, 400 ng/mL Ang-1, or 150 ng/mL IGF-1. Scale bar: 60 µm. **G:** Sprouting (% of control) of treated HUVEC spheroids depicted in (F), as assessed by spheroid sprouting assay (n = 15). Data are presented as means ± SEM. *P < 0.05, **P < 0.01, ***P < 0.001; ns, not significant.

To elucidate the molecular mechanisms underlying the anti-angiogenic effect of carvacrol, we initially examined the expression of several RTKs, i.e., VEGFR2, VEGFR1, EGFR, Tie2, FGFR1, and IGF1R, in HUVECs, given their pivotal role in angiogenesis regulation 5, 6. Western blot analyses showed that treatment with 200 µM carvacrol for 4 h significantly down-regulates the protein levels of VEGFR2, Tie2, FGFR1, and IGF1R in HUVECs without affecting VEGFR1 and EGFR expression (Fig. 2A, B). Of note, the carvacrol-induced RTKs down-regulation was observed as early as 2 h after treatment (Fig. S1). However, the identical dose of carvacrol induced no change in the mRNA levels of VEGFR2, Tie2, FGFR1, and IGF1R, as assessed by qRT-PCR assays (Fig. 2C). These findings suggest that carvacrol down-regulates these RTKs post-transcriptionally.

To determine whether carvacrol induces the degradation of VEGFR2, Tie2, FGFR1, and IGF1R, we pre-treated HUVECs with the proteasome inhibitor MG132 and the lysosome inhibitor CQ for 2 h before exposure to carvacrol, since ubiquitin-proteasome and autophagy-lysosome are the two primary degradation systems in eukaryotic cells 38. Western blot results showed that in the presence of MG132 or CQ, carvacrol fails to down-regulate VEGFR2, Tie2, and IGF1R, as evidenced by comparable protein levels between the inhibitor (+) carvacrol (-) groups and the inhibitor (+) carvacrol (+) groups (Fig. 2D, E). This indicates that carvacrol promotes the degradation of VEGFR2, Tie2, and IGF1R via both the ubiquitin-proteasome and autophagy-lysosome pathways. In contrast, FGFR1 regulation displayed a distinct pattern. A significant difference remained between the inhibitor (+) carvacrol (-) groups and inhibitor (+) carvacrol (+) groups, suggesting that inhibition of either pathway did not fully restore FGFR1 levels. Notably, CQ, but not MG132, significantly increased FGFR1 levels in the inhibitor (+) carvacrol (+) group compared to the inhibitor (-) carvacrol (+) group (Fig. 2D, E). These results suggest that carvacrol-induced FGFR1 down-regulation is at least partially mediated via the lysosomal pathway rather than the proteasomal system.

To investigate whether the down-regulation of VEGFR2, FGFR1, Tie2, and IGF1R contributes to the anti-angiogenic effect of carvacrol, carvacrol-treated HUVEC spheroids were stimulated with VEGF, bFGF, angiopoietin-1 (Ang-1), and insulin-like growth factor 1 (IGF-1), which are the cognate ligands for VEGFR2, FGFR1, Tie2, and IGF1R, respectively. The rationale behind this approach was that if carvacrol suppresses angiogenesis primarily by promoting RTKs degradation, stimulation with these ligands would activate the remaining receptors and compensate for their enhanced degradation. Spheroid sprouting assays demonstrated that stimulation with these ligands significantly rescue carvacrol-suppressed EC spheroid sprouting (Fig. 2F, G), supporting the hypothesis that the anti-angiogenic effects of carvacrol are indeed dependent on RTKs.

Collectively, these findings suggest that the degradation of VEGFR2, FGFR1, Tie2, and IGF1R contributes to the anti-angiogenic effect of carvacrol.

### Inhibition of TRPM7/Zn^2+^/mTOR signaling contributes to carvacrol-induced RTKs degradation in ECs

#### Carvacrol binds the VL site of TRPM7 and inhibits its channel activity

**Figure 3:**
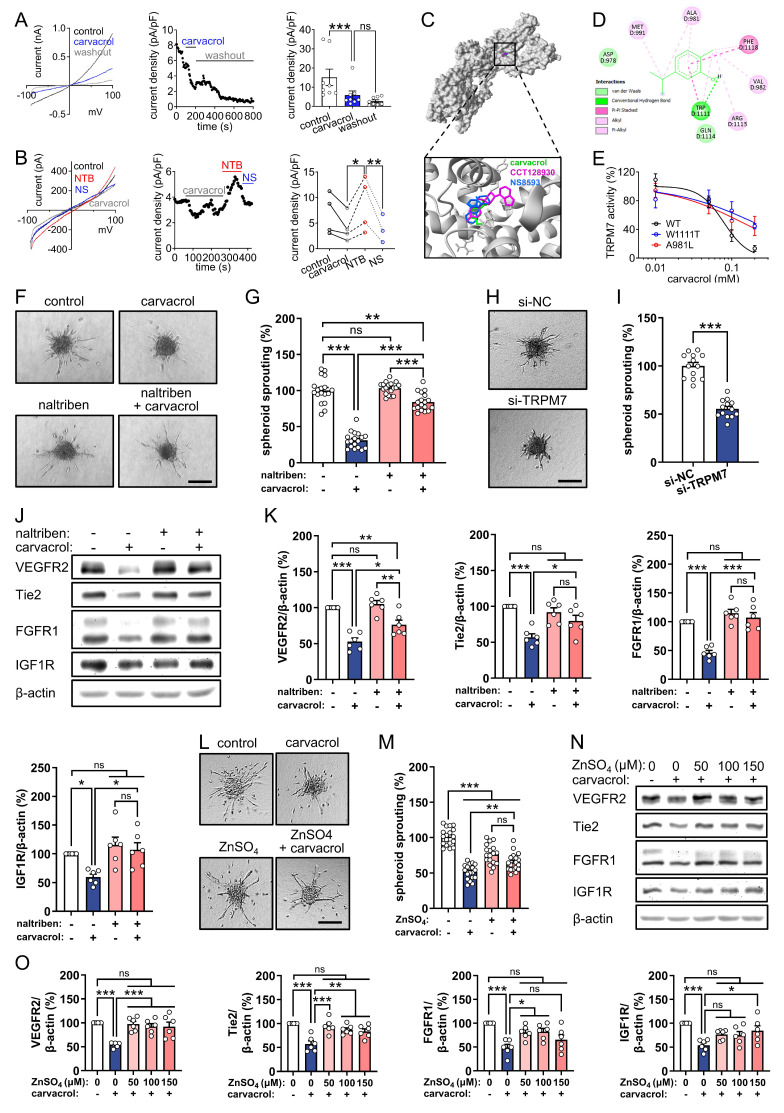
Carvacrol promotes RTKs degradation through blocking TRPM7-mediated Zn^2+^ influx in ECs. **A, B:** Left panels show representative current-voltage relationships of whole-cell membrane currents recorded from HUVECs exposed to 200 µM carvacrol followed by washout (A) or from HUVECs sequentially exposed to 200 µM carvacrol, 50 µM naltriben (NTB; TRPM7 channel activator), and 100 µM NS8593 (NS; TRPM7 channel inhibitor) (B). Middle panels show time courses of current density (pA/pF) measured at +80 mV in the HUVECs depicted in the left panels. Right panels show outward current densities (pA/pF) recorded at +80 mV (n = 8 cells in A; n = 3-4 cells in B). **C:** 3D binding mode of carvacrol (green) within the TRPM7 channel compared with reference ligands CCT128930 (magenta; PDB ID: 8W2L) and NS8593 (blue), as assessed by blind molecular docking. **D:** 2D interaction diagram showing the predicted hydrogen bonds and hydrophobic contacts between carvacrol and key TRPM7 residues, as assessed by blind molecular docking. **E:** Concentration-response curves illustrating the inhibition of WT and mutant TRPM7 (A981L and W1111A) channel currents by carvacrol, as assessed by the whole-cell patch-clamp measurement (n = 5-7 cells). **F:** Representative images of HUVEC spheroids that were treated for 24 h with 0.1% DMSO (vehicle) or 200 µM carvacrol in the absence or presence of 10 µM naltriben. Scale bar: 90 µm. **G:** Sprouting (% of control) of treated HUVEC spheroids depicted in (F), as assessed by spheroid sprouting assay (n = 18).** H:** Representative images of HUVEC spheroids that were cultured for 24 h. The cells were transfected with si-NC or si-TRPM7 for 48 h prior to the assay. Scale bar: 90 µm. **I:** Sprouting (% of si-NC) of transfected HUVEC spheroids depicted in (H), as assessed by spheroid sprouting assay (n = 13). **J:** Representative Western blots showing VEGFR2, Tie2, FGFR1, IGF1R, and β-actin expression in HUVECs that were pre-treated without or with 10 µM naltriben for 1 h and then exposed to 0.1% DMSO (vehicle) or 200 µM carvacrol for another 4 h. **K:** Expression levels (% of control) of VEGFR2, Tie2, FGFR1, and IGF1R normalized to β-actin in treated HUVECs depicted in (J), as assessed by Western blotting (n = 6 independent experiments).** L:** Representative images of HUVEC spheroids that were treated for 24 h with 0.1% DMSO (vehicle) or 200 µM carvacrol in the absence or presence of 150 µM ZnSO₄. Scale bar: 90 µm. **M:** Sprouting (% of control) of treated HUVEC spheroids depicted in (L), as assessed by spheroid sprouting assay (n = 18). **N:** Representative Western blots showing VEGFR2, Tie2, FGFR1, IGF1R, and β-actin expression in HUVECs that were pre-treated with 0, 50, 100, or 150 µM ZnSO₄ for 2 h and then exposed to 0.1% DMSO (vehicle) or 200 µM carvacrol for another 4 h. **O:** Expression levels (% of control) of VEGFR2, Tie2, FGFR1, and IGF1R normalized to β-actin in treated HUVECs depicted in (N), as assessed by Western blotting (n = 6 independent experiments). Data are presented as means ± SEM. *P < 0.05, **P < 0.01, ***P < 0.001; ns, not significant.

Previous studies have demonstrated the inhibitory effect of carvacrol on TRPM7-like currents in several cell types 31, 39-41, but its effects in ECs have not been investigated. We therefore evaluated TRPM7 channel activity in carvacrol-treated HUVECs using whole-cell patch-clamp recordings. Treatment with carvacrol markedly suppressed TRPM7-like currents in HUVECs, and this suppression persisted following washout (Fig. 3A). To determine whether carvacrol-induced current reduction specifically results from TRPM7 channel inhibition, we employed naltriben, a selective TRPM7 channel activator 42. Interestingly, naltriben fully reversed the carvacrol-induced current reduction. Furthermore, the naltriben-mediated current rescue was completely abolished by NS8593, a well-characterized TRPM7 channel inhibitor 43 (Fig. 3B). These results confirm that carvacrol inhibits TRPM7 channel activity in HUVECs.

We then performed unbiased molecular docking simulations to explore the potential binding modes of carvacrol within the TRPM7 channel using a C-terminally truncated mouse TRPM7 construct (PDB ID: 8W2L) that represents a functional channel lacking the kinase domain. Our results suggest that, similar to the known TRPM7 inhibitors CCT128930 and NS8593 43, 44, carvacrol preferentially binds to the vanilloid-like (VL) site of TRPM7, with a binding free energy of -5.04 kcal/mol (Fig. 3C). The predicted carvacrol-TRPM7 interactions were predominantly hydrophobic, with a single hydrogen bond formed between the hydroxyl group of carvacrol and the W1111 residue in the TRP helix (Fig. 3D). To experimentally validate the direct binding of carvacrol to the VL site, we introduced point mutations at key residues predicted to mediate carvacrol interactions (A981L and W1111A) and compared TRPM7 currents in HEK293T cells expressing wild-type (WT) or mutant channels. Concentration-response analyses revealed half-maximal inhibitory concentration (IC_50_) values of 78.3 µM for WT TRPM7, 135.4 µM for A981L, and 192.9 µM for W1111A mutants, corresponding to 1.7- and 2.5-fold increases, respectively (Fig. 3E). The fitted Hill coefficients (n_H_) were -2.72 for WT, -0.83 for A981L, and -0.91 for W1111A, indicating a marked reduction in the steepness of inhibition curves for both mutants. These findings provide strong functional evidence that carvacrol inhibits TRPM7 through direct interaction with the VL site, with residues A981 and W1111 serving as key molecular determinants of binding affinity and channel inhibition.

#### Carvacrol exerts anti-angiogenic activity by targeting the TRPM7/Zn^2+^/mTOR axis

In addition to acting as a TRPM7 channel inhibitor, carvacrol has also been identified as an activator of transient receptor potential ankyrin 1 (TRPA1), transient receptor potential vanilloid-3 (TRPV3), and transient receptor potential melastatin 8 (TRPM8) channels 45, 46. To determine whether TRPM7 specifically mediates the carvacrol-induced inhibition of angiogenesis, carvacrol-treated HUVEC spheroids were exposed to 2.5 µM A-967079 (TRPA1 antagonist), 150 µM 74a (TRPV3 antagonist), 5 µM AMTB (TRPM8 antagonist), and 10 µM naltriben (TRPM7 agonist). These moderately effective concentrations of each compound, determined by WST-1 assays (Fig. S2A-D), were utilized to avoid cytotoxicity. Interestingly, activation of the TRPM7 channel with naltriben most effectively reversed carvacrol-suppressed EC spheroid sprouting, although naltriben itself did not affect this process (Fig. 3F, G and Fig. S2E, F). Consistently, knockdown of TRPM7 using its specific siRNAs mimicked the inhibitory effect of carvacrol on HUVEC spheroid sprouting (Fig. 3H, I). Furthermore, we assessed the expression of VEGFR2, Tie2, FGFR1, and IGF1R in HUVECs that were pre-treated with naltriben before exposure to carvacrol for 4 h. Western blot analyses revealed that pre-treatment with naltriben markedly prevents the carvacrol-induced degradation of RTKs (Fig. 3J, K), suggesting that carvacrol induces RTKs degradation in HUVECs through inhibiting TRPM7 channel activity.

As an ion channel, TRPM7 critically mediates the cellular uptake of essential divalent cations, including Ca^2+^, Mg^2+^, and Zn^2+^ 21. Inhibition of TRPM7 channel activity consequently reduces the influx of these cations. To determine which cation mediates the anti-angiogenic activity of carvacrol, we exposed carvacrol-treated HUVEC spheroids to 5 mM CaCl₂, 2.5 mM MgSO₄, or 150 µM ZnSO₄. The doses of each cation used were determined as moderately effective concentrations by means of WST-1 assays (Fig. S3A-C). Our results showed that the supplementation of Zn^2+^, but not Ca^2+^ or Mg^2+^, prevents carvacrol from suppressing EC spheroid sprouting (Fig. 3L, M and Fig. S3D-G). Of note, CaCl₂, MgSO₄, or ZnSO₄ alone significantly inhibited this process (Fig. 3L, M and Fig. S3D-G). Moreover, pre-treatment with 50, 100, or 150 µM ZnSO₄ largely counteracted the carvacrol-induced degradation of VEGFR2, Tie2, FGFR1, and IGF1R in HUVECs (Fig. 3N, O). These findings indicate that inhibition of Zn^2+^ influx through the TRPM7 channel contributes to carvacrol-induced RTKs degradation and ultimate angiogenesis inhibition.

**Figure 4:**
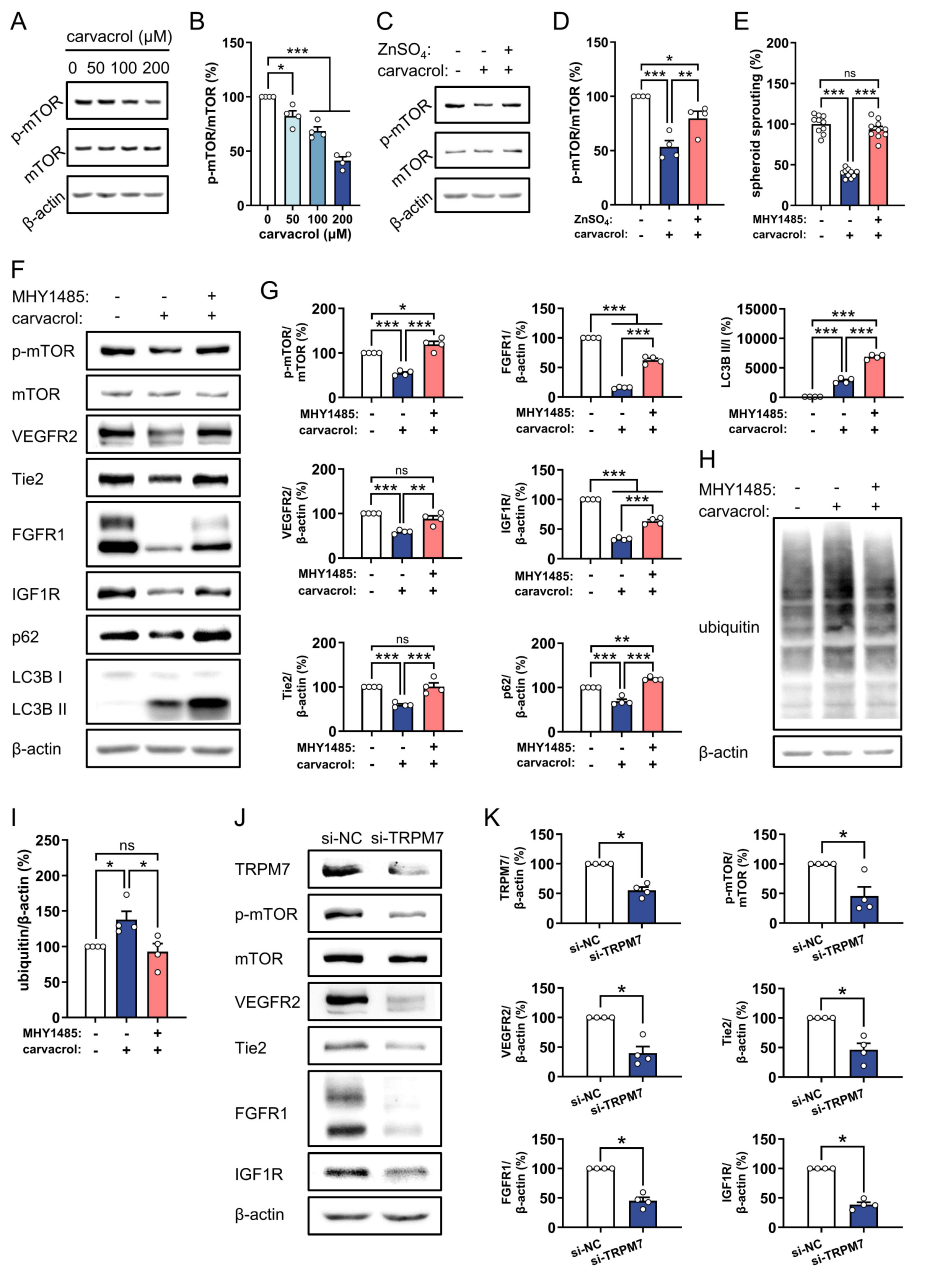
Carvacrol promotes RTKs degradation in ECs through inhibiting mTOR phosphorylation, downstream of TRPM7/Zn^2+^. **A:** Representative Western blots showing p-mTOR, mTOR, and β-actin expression in HUVECs that were treated for 4 h with 0, 50, 100, and 200 µM carvacrol. **B:** Expression levels (% of 0 µM) of p-mTOR normalized to mTOR in treated HUVECs depicted in (A), as assessed by Western blotting (n = 4 independent experiments). **C:** Representative Western blots showing p-mTOR, mTOR, and β-actin expression in HUVECs that were pre-treated without or with 50 µM ZnSO₄ for 2 h and then exposed to 0.1% DMSO (vehicle) or 200 µM carvacrol for another 4 h. **D:** Expression levels (% of control) of p-mTOR normalized to mTOR in treated HUVECs depicted in (C), as assessed by Western blotting (n = 4 independent experiments). **E:** Sprouting (% of control) of HUVEC spheroids treated for 24 h with 0.1% DMSO (vehicle) or 200 µM carvacrol in the absence or presence of 10 µM MHY1485 (mTOR activator), as assessed by spheroid sprouting assay (n = 10). **F:** Representative Western blots showing p-mTOR, mTOR, VEGFR2, Tie2, FGFR1, IGF1R, p62, LC3B, and β-actin expression in HUVECs that were pre-treated without or with 10 µM MHY1485 for 2 h and then exposed to 0.1% DMSO (vehicle) or 200 µM carvacrol for another 4 h. **G:** Expression levels (% of control) of p-mTOR normalized to mTOR, and VEGFR2, Tie2, FGFR1, IGF1R, and p62 normalized to β-actin, along with the LC3B II/I ratio in treated HUVECs depicted in (F), as assessed by Western blotting (n = 4 independent experiments). **H:** Representative Western blots showing ubiquitin and β-actin expression in HUVECs that were pre-treated without or with 10 µM MHY1485 for 1 h and then exposed to 0.1% DMSO (vehicle) or 200 µM carvacrol for another 1 h. **I:** Expression levels (% of control) of ubiquitin normalized to β-actin in treated HUVECs depicted in (H), as assessed by Western blotting (n = 4 independent experiments). **J:** Representative Western blots showing TRPM7, p-mTOR, mTOR, VEGFR2, Tie2, FGFR1, IGF1R, and β-actin expression in HUVECs that were transfected with si-NC or si-TRPM7 for 48 h. **K:** Expression levels (% of si-NC) of p-mTOR normalized to mTOR, and TRPM7, VEGFR2, Tie2, FGFR1, and IGF1R normalized to β-actin in treated HUVECs depicted in (J), as assessed by Western blotting (n = 4 independent experiments). Data are presented as means ± SEM. *P < 0.05, **P < 0.01, ***P < 0.001; ns, not significant.

Given that mTOR regulates protein degradation via both the ubiquitin-proteasome and autophagy-lysosome systems 47-49, we hypothesized that mTOR serves as a downstream effector of the TRPM7/Zn²⁺ axis, mediating RTKs degradation induced by carvacrol. Western blot analyses revealed that carvacrol dose-dependently reduces p-mTOR levels in HUVECs (Fig. 4A, B). This reduction was significantly reversed by Zn^2+^ supplementation (Fig. 4C, D), establishing mTOR as a downstream mediator of TRPM7/Zn²⁺. To determine whether the reduced mTOR phosphorylation contributes to carvacrol-induced angiogenesis suppression, HUVEC spheroids were treated with carvacrol in the presence of the mTOR activator MHY1485. Activation of mTOR with 10 µM MHY1485 completely abolished the inhibitory effect of carvacrol on EC spheroid sprouting (Fig. 4E), demonstrating that mTOR inhibition is required for carvacrol's anti-angiogenic effects. Moreover, pre-treatment with MHY1485 efficiently reversed carvacrol-induced down-regulation of p-mTOR, VEGFR2, Tie2, FGFR1, and IGF1R in HUVECs (Fig. 4F, G). MHY1485 also prevented the carvacrol-induced reduction in p62 levels, enhanced the carvacrol-induced increase in the LC3B II/I ratio (Fig. 4F, G), indicating that carvacrol promotes autophagy by inhibiting mTOR phosphorylation. In parallel, MHY1485 suppressed the carvacrol-induced accumulation of ubiquitinated proteins (Fig. 4H, I), demonstrating that this phytochemical also enhances protein ubiquitination via mTOR inhibition. These findings indicate that carvacrol promotes the degradation of angiogenesis-related RTKs through both the autophagy-lysosome and ubiquitin-proteasome pathways by down-regulating mTOR phosphorylation, ultimately resulting in suppressed angiogenesis. In addition, knockdown of TRPM7 in HUVECs significantly reduced the expression of p-mTOR, VEGFR2, Tie2, FGFR1, and IGF1R (Fig. 4J, K), phenocopying the effects of carvacrol treatment.

Taken together, these findings suggest that the blockade of TRPM7/Zn^2+^/mTOR signaling contributes to the carvacrol-induced degradation of VEGFR2, Tie2, FGFR1, and IGF1R in ECs, resulting in the inhibition of angiogenesis.

### Carvacrol inhibits VM in TNBC cells through promoting FGFR1 and IGF1R degradation

**Figure 5:**
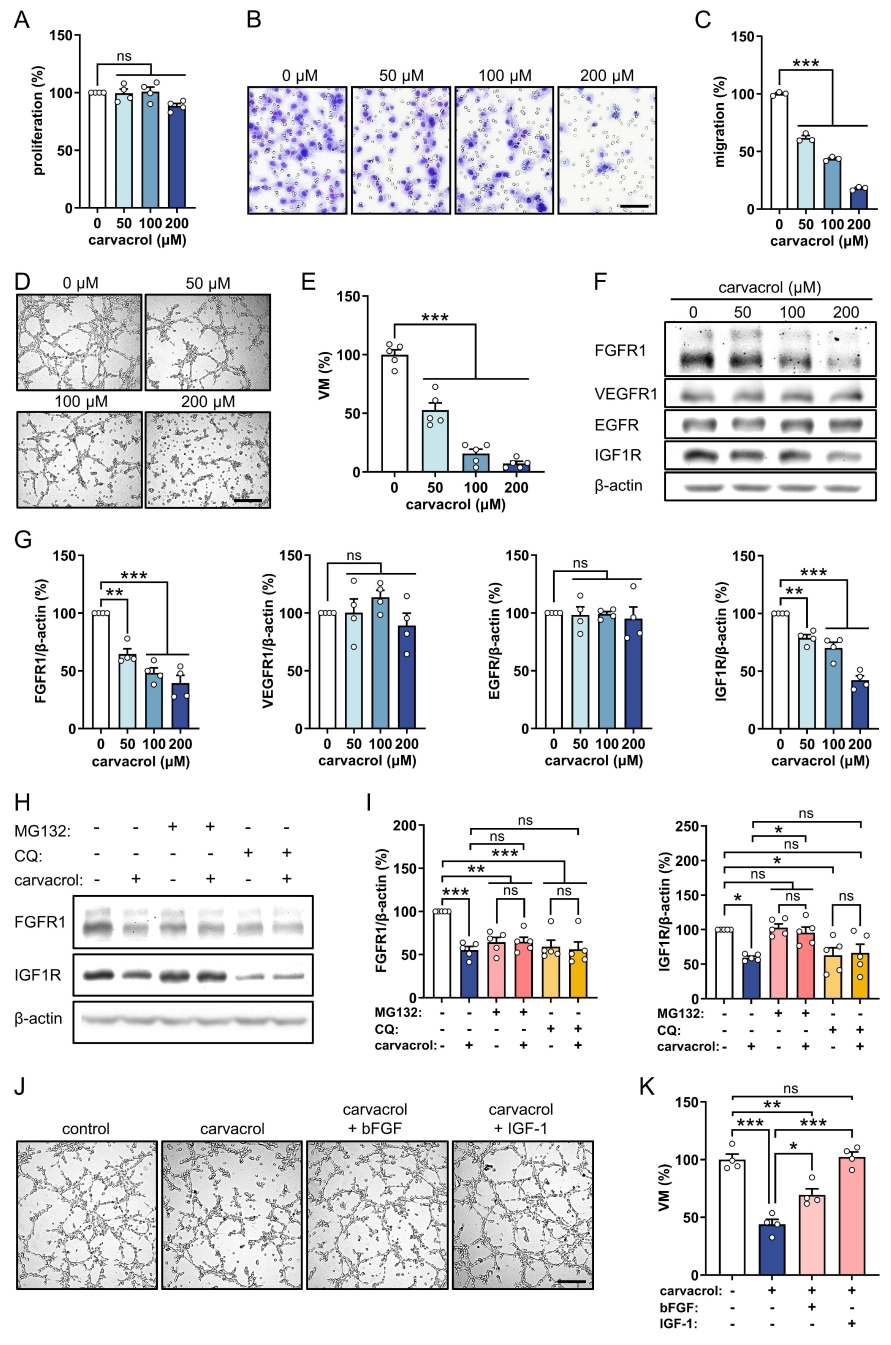
Carvacrol inhibits VM through promoting the degradation of FGFR1 and IGF1R in TNBC cells. **A:** Proliferation (% of 0 µM) of MDA-MB-231 cells that were treated for 24 h with 0, 50, 100, and 200 µM carvacrol, as assessed by BrdU incorporation assay (n = 4 independent experiments). **B:** Representative images of migrated MDA-MB-231 cells. The cells were treated with 0, 50, 100, and 200 µM carvacrol for 24 h prior to the assay. Scale bar: 63 µm. **C:** Migration (% of 0 µM) of treated MDA-MB-231 cells depicted in (B), as assessed by Transwell migration assay (n = 3). **D:** Representative images of tube-forming MDA-MB-231 cells that were treated for 18 h with 0, 50, 100, and 200 µM carvacrol. Scale bar: 260 µm. **E:** VM (% of 0 µM) in treated MDA-MB-231 cells depicted in (D), as assessed by tube formation assay (n = 5). **F:** Representative Western blots showing FGFR1, VEGFR1, EGFR, IGF1R, and β-actin expression in MDA-MB-231 cells that were treated for 4 h with 0, 50, 100, and 200 µM carvacrol. **G:** Expression levels (% of 0 µM) of FGFR1, VEGFR1, EGFR, and IGF1R normalized to β-actin in treated MDA-MB-231 cells depicted in (F), as assessed by Western blotting (n = 4 independent experiments). **H:** Representative Western blots showing FGFR1, IGF1R, and β-actin expression in MDA-MB-231 cells that were pre-treated without or with 10 µM MG132 (proteasome inhibitor) or 200 µM CQ (lysosome inhibitor) for 2 h and then exposed to 0.1% DMSO (vehicle) or 200 µM carvacrol for another 4 h. **I:** Expression levels (% of control) of FGFR1 and IGF1R normalized to β-actin in treated MDA-MB-231 cells depicted in (H), as assessed by Western blotting (n = 5 independent experiments). **J:** Representative images of tube-forming MDA-MB-231 cells that were treated for 18 h with 0.1% DMSO (vehicle) or 50 µM carvacrol in the absence or presence of 100 ng/mL bFGF or 150 ng/mL IGF-1. Scale bar: 200 µm. **K:** VM (% of control) of treated MDA-MB-231 cells depicted in (J), as assessed by tube formation assay (n = 4). Data are presented as means ± SEM. *P < 0.05, **P < 0.01, ***P < 0.001; ns, not significant.

We next investigated the impact of carvacrol on TNBC cells. Consistent with the results shown in Fig. 1B, 50, 100, and 200 µM carvacrol had no significant effect on the proliferation of MDA-MB-231 cells (Fig. 5A), although it significantly inhibited HUVEC proliferation as demonstrated in Fig. 1D. However, these concentrations of carvacrol significantly reduced the motility of MDA-MB-231 cells (Fig. 5B, C). More importantly, this phytochemical strongly inhibited VM in MDA-MB-231, HCC1937, and 4T1 cells in a dose-dependent manner, as demonstrated by tube formation assays (Fig. 5D, E and Fig. S4). Notably, this assay is the most widely used and well-established method for evaluating the vascular activity of tumor cells *in vitro* 50.

To understand the molecular mechanisms underlying the anti-VM effect of carvacrol, we assessed the expression of several RTKs, including VEGFR1, EGFR, FGFR1, and IGF1R, in MDA-MB-231 cells treated with different concentrations of carvacrol. Of note, VEGFR2 and Tie2 were not included in this assay because they could not be detected in MDA-MB-231 cells, as shown in our previous studies 36, 51. Western blot analyses revealed that carvacrol dose-dependently down-regulates FGFR1 and IGF1R in TNBC cells, but not VEGFR1 and EGFR (Fig. 5F, G). Furthermore, pre-treatment with the proteasome inhibitor MG132 or the lysosome inhibitor CQ hindered carvacrol from down-regulating FGFR1 and IGF1R, as evidenced by comparable protein levels between the inhibitor (+) carvacrol (-) groups and the inhibitor (+) carvacrol (+) groups (Fig. 5H, I). This indicates that carvacrol promotes the degradation of FGFR1 and IGF1R in TNBC cells through both the ubiquitin-proteasome and autophagy-lysosome pathways. In addition, activation of FGFR1 and IGF1R with their respective ligands, bFGF and IGF-1, significantly prevented carvacrol-suppressed VM in MDA-MB-231 cells (Fig. 5J, K). These findings indicate that degradation of FGFR1 and IGF1R contributes to the anti-VM activity of carvacrol in TNBC cells.

### Inhibition of TRPM7/Zn^2+^/mTOR signaling contributes to carvacrol-induced RTKs degradation in TNBC cells

**Figure 6:**
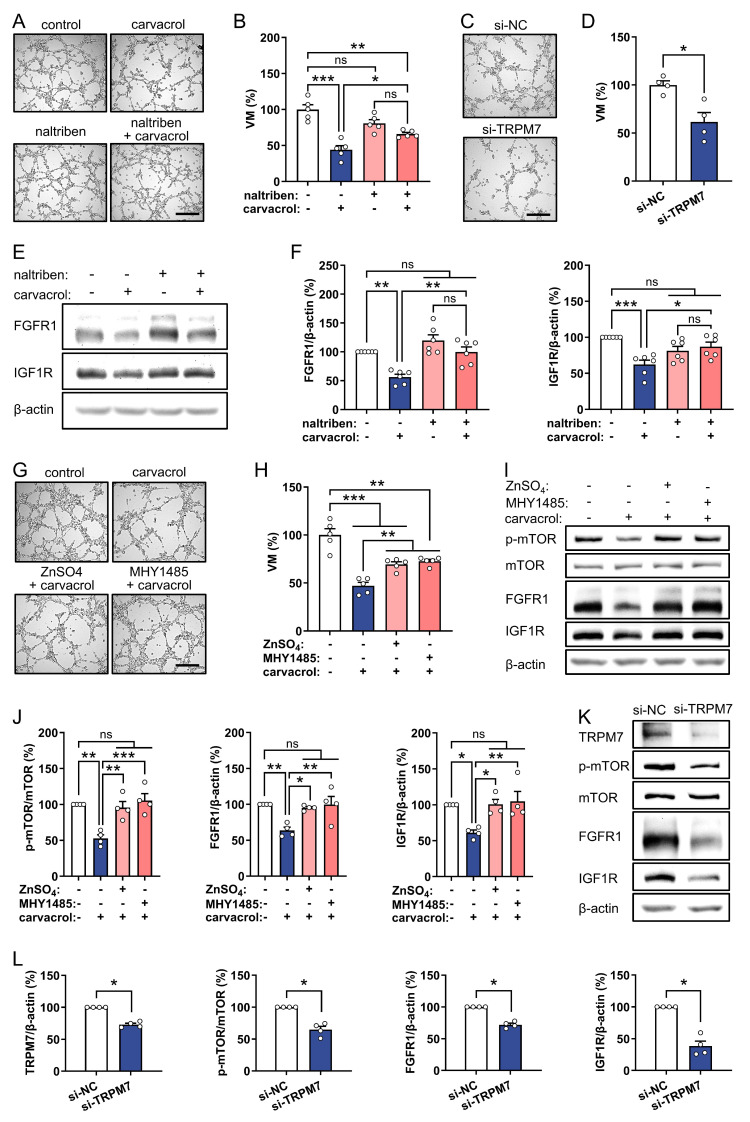
Carvacrol inhibits VM through blocking TRPM7/Zn^2+^/mTOR signaling in TNBC cells. **A:** Representative images of tube-forming MDA-MB-231 cells that were treated for 18 h with 0.1% DMSO (vehicle) or 50 µM carvacrol in the absence or presence of 10 µM naltriben (TRPM7 channel activator). Scale bar: 300 µm. **B:** VM (% of control) of treated MDA-MB-231 cells depicted in (A), as assessed by tube formation assay (n = 5). **C:** Representative images of tube-forming MDA-MB-231 cells that were cultured for 18 h. The cells were transfected with si-NC or si-TRPM7 for 48 h prior to the assay. Scale bar: 300 µm. **D:** VM (% of si-NC) of transfected MDA-MB-231 cells depicted in (C), as assessed by tube formation assay (n = 4). **E:** Representative Western blots showing FGFR1, IGF1R, and β-actin expression in MDA-MB-231 cells that were pre-treated without or with 10 µM naltriben for 1 h and then exposed to 0.1% DMSO (vehicle) or 200 µM carvacrol for another 4 h. **F:** Expression levels (% of control) of FGFR1 and IGF1R normalized to β-actin in treated MDA-MB-231 cells depicted in (E), as assessed by Western blotting (n = 6 independent experiments). **G:** Representative images of tube-forming MDA-MB-231 cells that were treated for 18 h with 0.1% DMSO (vehicle) or 50 µM carvacrol in the absence or presence of 50 µM ZnSO₄ or 10 µM MHY1485 (mTOR activator). Scale bar: 300 µm. **H:** VM (% of control) of treated MDA-MB-231 cells depicted in (G), as assessed by tube formation assay (n = 5). **I:** Representative Western blots showing p-mTOR, mTOR, FGFR1, IGF1R, and β-actin expression in MDA-MB-231 cells that were pre-treated without or with 50 µM ZnSO₄ or 10 µM MHY1485 for 2 h and then exposed to 0.1% DMSO (vehicle) or 200 µM carvacrol for another 4 h.** J:** Expression levels (% of control) of p-mTOR normalized to mTOR, and FGFR1 and IGF1R normalized to β-actin in treated MDA-MB-231 cells depicted in (I), as assessed by Western blotting (n = 4 independent experiments). **K:** Representative Western blots showing TRPM7, p-mTOR, mTOR, FGFR1, IGF1R, and β-actin expression in MDA-MB-231 cells that were transfected with si-NC or si-TRPM7 for 48 h. **L:** Expression levels (% of si-NC) of p-mTOR normalized to mTOR, and TRPM7, FGFR1, and IGF1R normalized to β-actin in transfected MDA-MB-231 cells depicted in (K), as assessed by Western blotting (n = 4 independent experiments). Data are presented as means ± SEM. *P < 0.05, **P < 0.01, ***P < 0.001; ns, not significant.

To investigate whether TRPM7 channel inhibition also mediates the inhibitory effect of carvacrol on VM, we treated MDA-MB-231 cells with vehicle or 50 µM carvacrol in the presence of naltriben. Tube formation assays revealed that activation of the TRPM7 channel with naltriben effectively reverses carvacrol-suppressed VM in MDA-MB-231 cells, although naltriben itself had no effect on this process (Fig. 6A, B). Consistently, TRPM7 knockdown mimicked the inhibitory effect of carvacrol on VM (Fig. 6C, D). Furthermore, pre-treatment with naltriben for 1 h completely prevented the carvacrol-induced degradation of FGFR1 and IGF1R in MDA-MB-231 cells (Fig. 6E, F). Additionally, we added ZnSO_4_ or MHY1485 to carvacrol-treated MDA-MB-231 cells and observed that 50 and 100 µM ZnSO_4_ or 10 µM MHY1485 significantly reversed the carvacrol-induced inhibition of VM (Fig. 6G, H and Fig. S5A, B). These treatments also counteracted the down-regulation of mTOR phosphorylation and the degradation of FGFR1 and IGF1R (Fig. 6I, J and Fig. S5C-E). Importantly, TRPM7 knockdown recapitulated the carvacrol-induced reduction in p-mTOR, FGFR1, and IGF1R expression in TNBC cells (Fig. 6K, L). Taken together, these findings suggest that inhibition of TRPM7/Zn^2+^/mTOR signaling contributes to the carvacrol-induced degradation of FGFR1 and IGF1R in TNBC cells, resulting in the inhibition of VM.

### Carvacrol inhibits TNBC vascularization and growth in a dorsal skinfold chamber model

**Figure 7:**
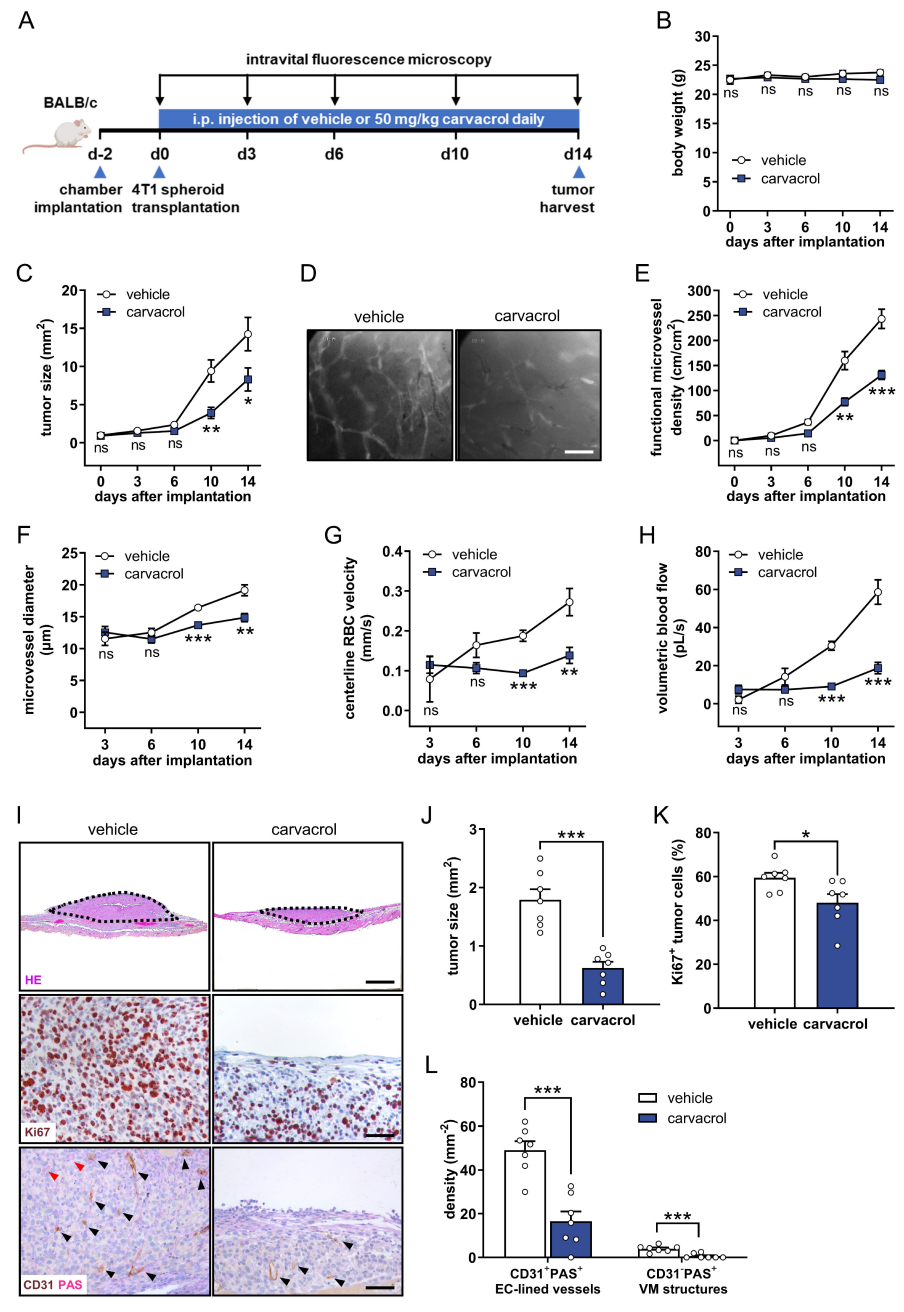
Carvacrol inhibits tumor vascularization and growth in the dorsal skinfold chamber model. **A:** Schematic timeline of the dorsal skinfold chamber model. **B:** Body weight (g) of vehicle- and carvacrol-treated mice on days 0, 3, 6, 10, and 14 after spheroid transplantation (n = 7). **C:** Size (mm^2^) of 4T1 tumors in vehicle- and carvacrol-treated mice on days 0, 3, 6, 10, and 14 after spheroid transplantation, as assessed by intravital fluorescence microscopy (n = 7). **D:** Representative images of newly formed microvessels in vehicle- and carvacrol-treated 4T1 tumors on day 14 after spheroid transplantation. Scale bar: 150 µm. **E:** Functional microvessel density (cm/cm^2^) of 4T1 tumors in vehicle- and carvacrol-treated mice on days 0, 3, 6, 10, and 14 after spheroid transplantation, as assessed by intravital fluorescence microscopy (n = 7). **F-H:** Diameter (μm; F), centerline RBC velocity (mm/s; G), and volumetric blood flow (pL/s; H) of tumor microvessels in vehicle- and carvacrol-treated mice, as assessed by intravital fluorescence microscopy (n = 7). **I:** Representative images of HE-stained (dotted line = tumor border), Ki67-stained, and CD31 and PAS double-stained sections of 4T1 tumors from vehicle- and carvacrol-treated mice on day 14 after spheroid transplantation. Scale bars: 200 μm (upper panel) and 55 μm (middle and lower panels). **J:** Size (mm^2^) of 4T1 tumors depicted in (I), as assessed by HE staining (n = 7). **K:** Ki67^+^ tumor cells (% of total cell number) in 4T1 tumors depicted in (I), as assessed by immunohistochemical staining of Ki67 (n = 7). **L:** Density (mm^-2^) of CD31^+^PAS^+^ EC-lined vessels (black arrowheads) and CD31^-^PAS^+^ VM structures (red arrowhead) in 4T1 tumors depicted in (I), as assessed by CD31 and PAS double staining (n = 7). Data are presented as means ± SEM. *P < 0.05, **P < 0.01, ***P < 0.001; ns, not significant.

To repeatedly evaluate the effects of carvacrol on TNBC vascularization and growth, we used a mouse dorsal skinfold chamber model. For this purpose, spheroids of murine 4T1 cells were transplanted into the dorsal skinfold chamber of syngeneic BALB/c mice 2 days after chamber implantation. Subsequently, the tumors in both vehicle- and carvacrol-treated mice were monitored via intravital fluorescence microscopy twice a week (Fig. 7A). Of note, daily intraperitoneal injections of carvacrol (50 mg/kg body weight) over 2 weeks were well tolerated, as indicated by comparable body weights of carvacrol- and vehicle-treated mice (Fig. 7B). In contrast, carvacrol treatment resulted in a significant reduction in tumor size on days 10 and 14 after spheroid transplantation when compared to controls (Fig. 7C). Moreover, carvacrol markedly reduced the density of functional microvessels with blood flow on days 10 and 14 after spheroid transplantation (Fig. 7D, E). Additional analyses of microhemodynamic parameters revealed a significant reduction in the diameter, centerline RBC velocity, and volumetric blood flow of tumor microvessels in the carvacrol group on days 10 and 14 after spheroid transplantation (Fig. 7F-H).

At the end of the experiments, tumors were harvested and processed for histological and immunohistochemical analyses. HE staining showed that carvacrol-treated tumors were markedly smaller in size (Fig. 7I, J). Moreover, carvacrol treatment significantly reduced the percentage of Ki67^+^ proliferating tumor cells (Fig. 7I, K), but induced no change in the percentage of cleaved caspase-3^+^ apoptotic tumor cells (Fig. S6A, B). In addition, CD31 and PAS double staining revealed that carvacrol significantly reduces the density of both CD31^+^ PAS^+^ EC-lined vessels and CD31^-^ PAS^+^ VM structures (Fig. 7I, L), suggesting its dual inhibition of angiogenesis and VM in the dorsal skinfold chamber model.

### Carvacrol inhibits TNBC vascularization and growth in an orthotopic xenograft model

**Figure 8:**
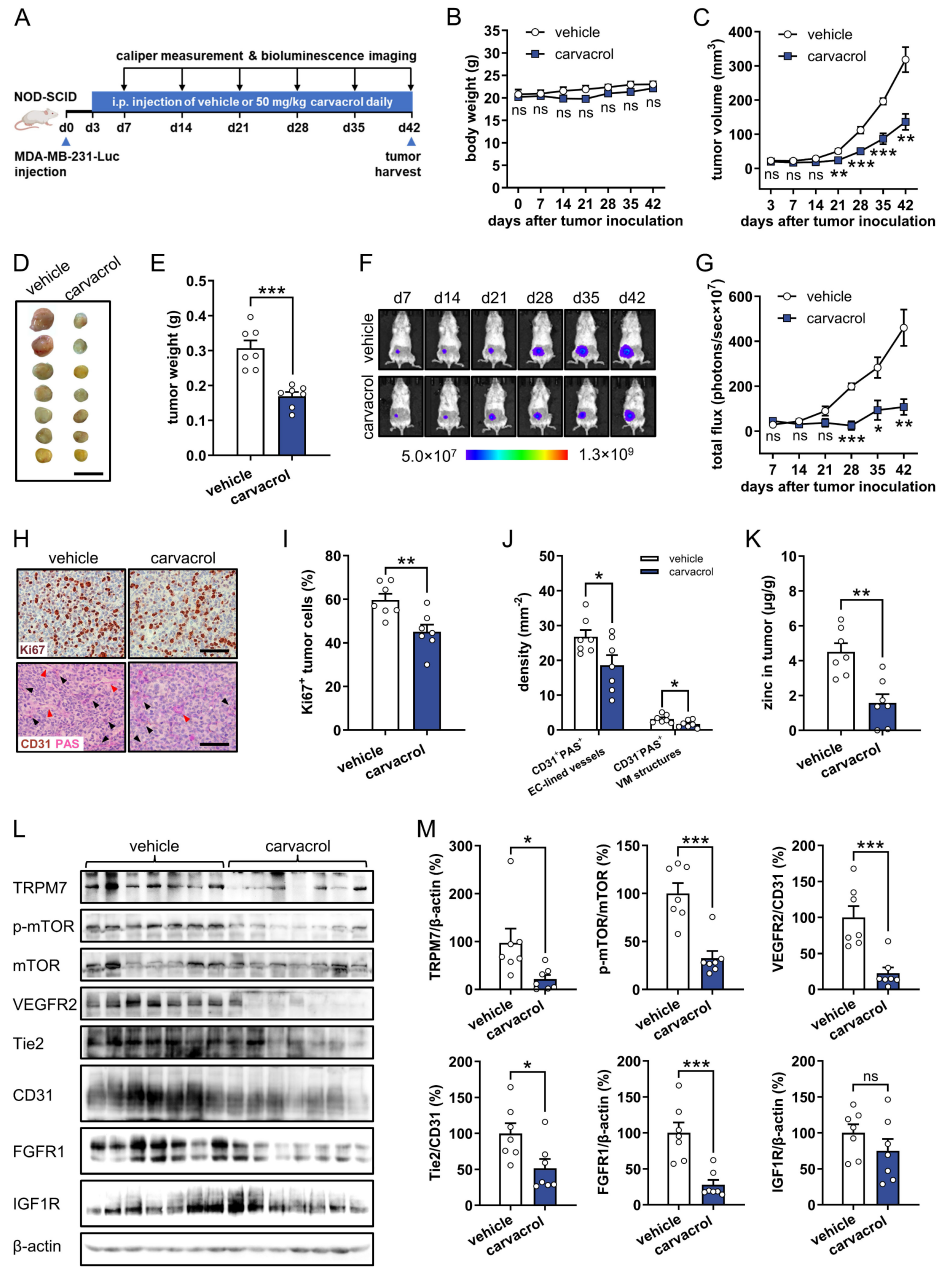
Carvacrol inhibits tumor vascularization and growth in the orthotopic xenograft model. **A:** Schematic timeline of the orthotopic xenograft model. **B:** Body weight (g) of vehicle- and carvacrol-treated mice on days 0, 7, 14, 21, 28, 35, and 42 after tumor inoculation (n = 7). **C:** Volume (mm^3^) of MDA-MB-231 tumors in vehicle- and carvacrol-treated mice on days 3, 7, 14, 21, 28, 35, and 42 after tumor inoculation, as assessed by caliper measurement (n = 7). **D:** Images of MDA-MB-231 tumors from vehicle- and carvacrol-treated mice on day 42 after tumor inoculation. Scale bar: 13 mm. **E:** Weight (g) of MDA-MB-231 tumors from vehicle- and carvacrol-treated mice on day 42 after tumor inoculation (n = 7). **F:** Representative bioluminescent images of tumors in vehicle- and carvacrol-treated mice on days 7, 14, 21, 28, 35, and 42 after tumor inoculation. **G:** Total flux (photons/sec×10^7^) of bioluminescent signals emitted from tumors depicted in (F), as assessed by bioluminescence imaging (n = 7). **H:** Representative images of Ki67-stained and CD31 and PAS double-stained sections of MDA-MB-231 tumors from vehicle- and carvacrol-treated mice on day 42 after tumor inoculation. Scale bars: 80 μm. **I:** Ki67^+^ tumor cells (% of total cell number) in MDA-MB-231 tumors depicted in (H), as assessed by immunohistochemical staining of Ki67 (n = 7). **J:** Density (mm^-2^) of CD31^+^PAS^+^ EC-lined vessels (black arrowheads) and CD31^-^PAS^+^ VM structures (red arrowheads) in MDA-MB-231 tumors depicted in (H), as assessed by CD31 and PAS double staining (n = 7).** K:** Zn^2+^ in MDA-MB-231 tumors (μg/g) from vehicle- and carvacrol-treated mice, as assessed by Zn^2+^ quantification assay (n = 7). **L:** Western blots showing TRPM7, p-mTOR, mTOR, VEGFR2, Tie2, CD31, FGFR1, IGF1R, and β-actin expression in tumors from vehicle- and carvacrol-treated mice. **M:** Expression levels (% of vehicle) of TRPM7 normalized to β-actin, p-mTOR normalized to mTOR, and VEGFR2 and Tie2 normalized to CD31, as well as FGFR1 and IGF1R normalized to β-actin in tumors from vehicle- and carvacrol-treated mice, as assessed by Western blotting (n = 7). Data are presented as means ± SEM. *P < 0.05, **P < 0.01, ***P < 0.001; ns, not significant.

We finally employed an orthotopic xenograft mouse model to assess the long-term effects of carvacrol on human TNBC progression. For this purpose, MDA-MB-231-Luc cells, a highly aggressive human TNBC cell line, were inoculated into the mammary fat pad of immunodeficient NOD-SCID mice. Thereafter, tumor growth in both vehicle- and carvacrol-treated mice was analyzed weekly by caliper measurements and bioluminescence imaging (Fig. 8A). Daily administration of 50 mg/kg body weight carvacrol from day 3 to day 42 after tumor inoculation had no significant impact on the body weight of the treated mice (Fig. 8B). However, carvacrol treatment significantly decreased the tumor volume starting from day 21 after tumor inoculation, as assessed by caliper measurements (Fig. 8C). At the end of the experiment on day 42, the orthotopic tumors were excised and weighed, revealing that tumors in carvacrol-treated mice exhibit reduced size and weight (Fig. 8D, E). Moreover, bioluminescence imaging demonstrated a marked reduction in the total flux of bioluminescent signals emitted from tumors in the carvacrol group on days 28, 35, and 42 after tumor inoculation (Fig. 8F, G).

Immunohistochemical analyses showed that administration of carvacrol significantly reduces the percentage of Ki67^+^ proliferating tumor cells (Fig. 8H, I) without affecting cleaved caspase-3^+^ apoptotic tumor cells (Fig. S6C, D). Furthermore, carvacrol markedly decreased the density of both CD31^+^ PAS^+^ EC-lined vessels and CD31^-^ PAS^+^ VM structures (Fig. 8H, J), indicating its dual inhibition of angiogenesis and VM in the orthotopic xenograft model. Moreover, carvacrol treatment significantly reduced intratumoral Zn²⁺ levels (Fig. 8K). Finally, Western blot analyses of tumor tissues demonstrated reduced expression of TRPM7, p-mTOR, VEGFR2, Tie2, and FGFR1 in the tumors from carvacrol-treated mice (Fig. 8L, M).

Taken together, these findings indicate that carvacrol suppresses TNBC vascularization and growth through sustained inhibition of the TRPM7/Zn^2+^/mTOR/RTKs signaling axis *in vivo*.

## Discussion

Carvacrol, a natural phenolic compound, has gained significant scientific interest over the past two decades due to its wide range of biological properties, including anti-tumor, antifungal, antiviral, and anti-inflammatory activities 17, 18. Its effective anti-tumor action has been mainly attributed to its anti-proliferative and cytotoxic effects through the induction of oxidative stress, inhibition of cell cycle progression, and initiation of apoptosis in various types of tumor cells 19, 52. However, although tumor vascularization is essential for tumor growth, the potential impact of carvacrol on this process remains uninvestigated. This knowledge gap is addressed by the present study, which reveals that compared to its minimal anti-proliferative effects on TNBC cells, carvacrol preferentially inhibits both angiogenesis in ECs and VM in TNBC cells, ultimately suppressing TNBC growth. These effects are mediated through targeted inhibition of the TRPM7/Zn^2+^/mTOR/RTKs signaling axis. In fact, this study provides the first evidence of a direct interaction between carvacrol and the VL site of TRPM7.

We first analyzed the anti-angiogenic effects of carvacrol on HUVECs, which are human primary macrovascular ECs extensively utilized for *in vitro* angiogenesis assays 53. Our results demonstrated a pleiotropic effect of carvacrol in inhibiting all key angiogenic activities of HUVECs, including proliferation, migration, tube formation, and spheroid sprouting. Subsequently, we confirmed the anti-angiogenic effect of carvacrol in an *ex vivo* aortic ring assay involving mouse aortic ECs, as well as in an *in vivo* Matrigel plug assay involving mouse microvascular ECs. The fact that carvacrol exerted suppressive effects on ECs from different species and tissue origins indicates the validity and reliability of our findings.

Moreover, we observed that carvacrol efficiently inhibits VM in two human TNBC cell lines, i.e., MDA-MB-231 and HCC1937, as well as in the mouse TNBC 4T1 cell line. We specifically focused on TNBC due to the urgent need for effective therapies against this aggressive subtype, in which VM is highly prevalent and contributes to tumor aggressiveness and anti-angiogenic resistance 54, 55. While carvacrol's anti-cancer properties have been reported, its effects on VM in TNBC remain unknown. Our findings establish carvacrol as a novel VM inhibitor in TNBC, expanding the repertoire of natural compounds with anti-VM activity.

Mechanistic investigations indicated that carvacrol inhibits angiogenesis through promoting the degradation of VEGFR2, Tie2, FGFR1, and IGF1R in HUVECs. VEGFR2, predominantly expressed in vascular ECs, plays a central role in coordinating VEGF-induced angiogenesis 56. Tie2, similarly mainly expressed in the vascular endothelium, stimulates EC survival, migration, tube formation, and sprouting upon activation 57. FGFR1, another significant RTK, can be activated by FGF1 and bFGF, triggering the angiogenic activity of ECs *in vitro* and *in vivo* 58. IGF1R, primarily activated by IGF1, mediates EC migration and tube formation 59. The combined blockade of these pivotal RTKs involved in angiogenesis may explain the high anti-angiogenic efficiency of carvacrol. However, unlike traditional anti-angiogenic RTK inhibitors blocking the kinase activity of receptors 5, carvacrol induces the degradation of RTKs via both the ubiquitin-proteasome and autophagy-lysosome systems. This mode of action has the potential to yield more complete and lasting inactivation of downstream signaling, thereby preventing the development of drug resistance and improving treatment outcomes in cancer patients 60, 61.

Similar to its anti-angiogenic action, carvacrol inhibited VM by triggering FGFR1 and IGF1R degradation in TNBC cells. Of note, this dual targeting effect of carvacrol differs significantly from current anti-angiogenic agents, which fail to inhibit VM and even accelerate its formation in TNBC 12, 13, suggesting that carvacrol may overcome key resistance mechanisms associated with these anti-angiogenic therapies. All these findings position carvacrol as a promising therapeutic candidate for the treatment of TNBC. So far, several compounds have been reported to inhibit VM by targeting VEGF, HER2, VE-cadherin, matrix metalloproteinases, hypoxia-inducible factor (HIF)-1α, among others 10. Our findings now suggest FGFR1 and IGF1R as novel targets for VM inhibition. However, the mechanisms by which FGFR1 and IGF1R drive VM need to be further clarified.

Additional mechanistic investigations suggested that inhibition of TRPM7/Zn^2+^/mTOR signaling causes the degradation of RTKs, mediating the anti-angiogenic and anti-VM effects of carvacrol. TRPM7 is ubiquitously expressed and significantly involved in various physiological and pathological processes, such as cancer 22, 62. Nevertheless, the role of TRPM7 in regulating angiogenesis is controversial. Several studies have reported that genetic knockdown or pharmacological inhibition of TRPM7 enhances the proliferation, migration, and tube formation of HUVECs 63-66. In contrast, Wu *et al.* recently demonstrated that TRPM7 knockdown suppresses HUVEC growth and tube formation 67. Our findings align with the latter study, demonstrating that inhibition of TRPM7 channel activity by carvacrol or TRPM7 knockdown effectively suppresses the angiogenic activities of HUVECs. These results help resolve the controversy by providing additional evidence supporting TRPM7's pro-angiogenic role. Importantly, we provide the first evidence for the involvement of TRPM7 in VM in TNBC, expanding our understanding of its channel's role in tumor vascularization beyond traditional angiogenesis. Additionally, previous studies have shown that TRPM7 inactivation facilitates the degradation of HIF-1α in ovarian cancer cells 68, regulates Zn^2+^-dependent HER2 expression in different cancer cells 69, whereas TRPM7 overexpression prevents MDMX degradation by modulating Zn^2+^ concentrations in human breast cancer MCF-7 cells 70. These findings, together with our current observations that carvacrol induces RTKs degradation by inhibiting TRPM7/Zn^2+^, suggest that TRPM7 plays a crucial role in maintaining the stability of key proteins involved in tumor progression. Therefore, TRPM7 represents a valuable therapeutic target for cancer treatment, offering a dual opportunity to simultaneously interfere with both tumor cell behavior and EC angiogenic activity. Of note, besides TRPM7, carvacrol has been reported to modulate several other TRP channels, including TRPA1, TRPV3, and TRPM8 45, 46. While our pharmacological inhibition and genetic knockdown data provide strong evidence that TRPM7 is the primary mediator of carvacrol's anti-vascular effects in our models, the potential contribution of these other channels to the broader anti-cancer activity of carvacrol in TNBC cannot be excluded. Future studies utilizing specific genetic knockouts or selective inhibitors of these channels would be required to fully delineate their individual contributions to the pleiotropic anti-cancer effects of carvacrol. mTOR is a well-known autophagy inhibitor that blocks autophagy-lysosome-mediated protein degradation 71. However, its role in ubiquitin-proteasome-mediated degradation is controversial, as mTOR has been shown to either up-regulate or down-regulate this process 47-49. Our study showed that mTOR inhibition by carvacrol promotes both the lysosomal and proteasomal degradation of several RTKs, suggesting an inhibitory effect of mTOR on both degradation pathways. However, the precise mechanism by which carvacrol-induced intracellular Zn^2+^ depletion leads to reduced mTOR phosphorylation needs further investigation. We propose that Zn^2+^ may directly interact with mTOR, thereby modulating its phosphorylation by altering local protein conformation or kinase accessibility. This notion is supported by previous evidence demonstrating that Zn^2+^ enhances mTOR activity in a cell-free protein kinase assay 72.

Finally, we evaluated the effects of carvacrol on TNBC progression in a dorsal skinfold chamber model and an orthotopic xenograft model. The dorsal skinfold chamber model serves as a valuable tool for the repeated analysis of tumor vascularization and growth *in vivo* 73. In this model, carvacrol administration began on the same day as 4T1 spheroid transplantation and continued for only 2 weeks to minimize animal discomfort. This short-term treatment with carvacrol significantly inhibited angiogenesis, VM, and growth of 4T1 tumors, highlighting its preventive potential against TNBC. In contrast, in the orthotopic xenograft model, carvacrol administration started 3 days after the injection of MDA-MB-231 cells, when the tumors became detectable, and ended 6 weeks after tumor inoculation. This long-term carvacrol treatment effectively suppressed angiogenesis, VM, and growth of MDA-MB-231 tumors, underscoring its therapeutic potential against TNBC. The combination of the dorsal skinfold chamber model of mouse TNBC and the orthotopic xenograft model of human TNBC offers a comprehensive and robust preclinical assessment of the efficacy of carvacrol in preventing and treating TNBC. Together with its natural origin and unique capacity to preferentially target both angiogenesis and VM, these *in vivo* findings position carvacrol as a promising candidate for clinical translation. This translational potential is further supported by carvacrol's Generally Recognized as Safe status established by the FDA for use as a flavoring agent 74, coupled with our *in vivo* findings showing no significant toxicity at therapeutic doses. Focusing on oral administration, toxicity studies reported an LD_50_ of 810 mg/kg in rats 17. While high-dose administration requires monitoring for potential hepatotoxicity, therapeutic doses (25-100 mg/kg) via oral gavage frequently induce hepatoprotective effects in mice 75, 76. Despite this favorable safety profile, carvacrol's systemic bioavailability is often constrained by high volatility and extensive first-pass metabolism, resulting in a short plasma half-life in various animal models 77. Therefore, the development of optimized delivery systems or the synthesis of analogs to enhance carvacrol's pharmacokinetic stability remains a strategic priority for its further therapeutic application.

**Figure 9:**
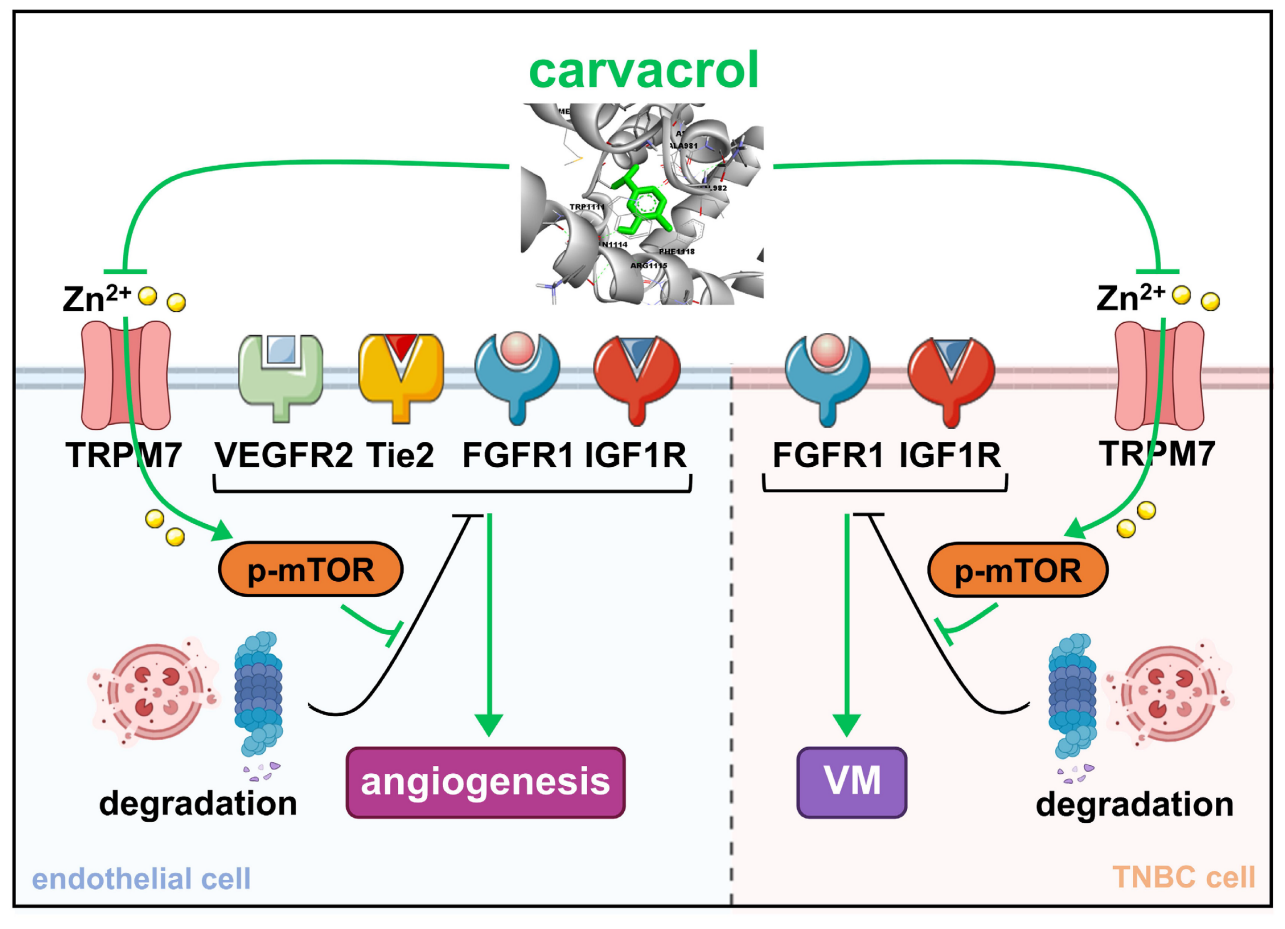
Molecular mechanisms of carvacrol's dual inhibitory effects on angiogenesis and VM. Carvacrol directly binds to the VL site of TRPM7, suppressing channel activity and reducing Zn²⁺ influx into the cytoplasm. This attenuates mTOR phosphorylation and promotes the proteasomal and lysosomal degradation of VEGFR2, Tie2, FGFR1, and IGF1R in ECs, as well as FGFR1 and IGF1R in TNBC cells, ultimately suppressing angiogenesis and VM, respectively.

In summary, our study reveals that carvacrol, a natural food additive, exerts dual anti-angiogenic and anti-VM effects by inhibiting the TRPM7/Zn^2+^/mTOR/RTKs signaling pathway in ECs and TNBC cells, respectively (Fig. 9). This leads to effective suppression of TNBC vascularization and growth. Thus, carvacrol represents a promising candidate for TNBC prevention and treatment, with potential as a therapeutic agent for other cancer types resistant to anti-angiogenic therapies. Additionally, this study establishes the TRPM7 channel as a novel anti-vascular target that regulates both angiogenesis and VM in TNBC.

## Supplementary Material

Supplementary figures.

## Data Availability

All data generated or analyzed during this study are included in this published article and its supplementary information file.

## Abbreviations

Ang-1: Angiopoietin-1
BrdU: Bromodeoxyuridine
CQ: Chloroquine diphosphate salt
DMEM: Dulbecco's modified Eagle's medium
EBM: Endothelial Cell Basal Medium
ECs: Endothelial cells
EGFR: Epidermal growth factor receptor
FCS: Fetal calf serum
FGFR: Fibroblast growth factor receptor
GAPDH: Glyceraldehyde-3-phosphate dehydrogenase
HDMECs: Human dermal microvascular endothelial cells
HE: Hematoxylin and eosin
HER2: Human epidermal growth factor receptor 2
HIF-1α: Hypoxia-inducible factor-1α
HUVECs: Human umbilical vein endothelial cells
IGF1R: Insulin-like growth factor 1 receptor
LDH: Lactate dehydrogenase
mRNA: Messenger RNA
mTOR: Mammalian target of rapamycin
PAS: Periodic acid-Schiff
qRT-PCR: Quantitative real-time polymerase chain reaction
RTKs: Receptor tyrosine kinases
TNBC: Triple-negative breast cancer
TRPA1: Transient receptor potential ankyrin 1
TRPM7: Transient receptor potential melastatin 7
TRPM8: Transient receptor potential melastatin 8
TRPV3: Transient receptor potential vanilloid-3
VEGF: Vascular endothelial growth factor
VEGFR: Vascular endothelial growth factor receptor
VM: Vasculogenic mimicry
