# Supplementary material for: The TRPM7 inhibitor carvacrol suppresses angiogenesis and vasculogenic mimicry in triple-negative breast cancer
Source: Int J Biol Sci. 2026 May 18;22(11):5634–58. doi: 10.7150/ijbs.130027 (PMC13282705; doi:10.7150/ijbs.130027)
Supplement: Supplementary file 1 — Supplementary figures. [file ijbsv22p5634s1.pdf]

Supplementary Material

The TRPM7 inhibitor carvacrol suppresses angiogenesis and vasculogenic mimicry in triple-negative breast cancer

Tianci Tang[1], Na Zhao[2], Moqin Qiu[1,4], Luisa Müller[1], Michael D. Menger[1], Vladimir Chubanov[3], Thomas Gudermann[3], Gabriela Krasteva-Christ[2], Matthias W. Laschke[1], Yuan Gu[1]*

[1]Institute for Clinical and Experimental Surgery, Saarland University, PharmaScienceHub (PSH), 66421 Homburg, Germany.

[2]Institute of Anatomy and Cell Biology, Saarland University, 66421 Homburg, Germany.

[3]Walther-Straub Institute of Pharmacology and Toxicology, LMU Munich, 80336 Munich, Germany.

[4]Department of Respiratory Oncology, Guangxi Medical University Cancer Hospital, 530021 Nanning, China.

***Corresponding author:**

Yuan Gu, PhD
Institute for Clinical and Experimental Surgery
Saarland University
66421 Homburg
Germany

phone: +49 6841 162 6368
fax: +49 6841 162 6553
e-mail: yuan.gu@uks.eu

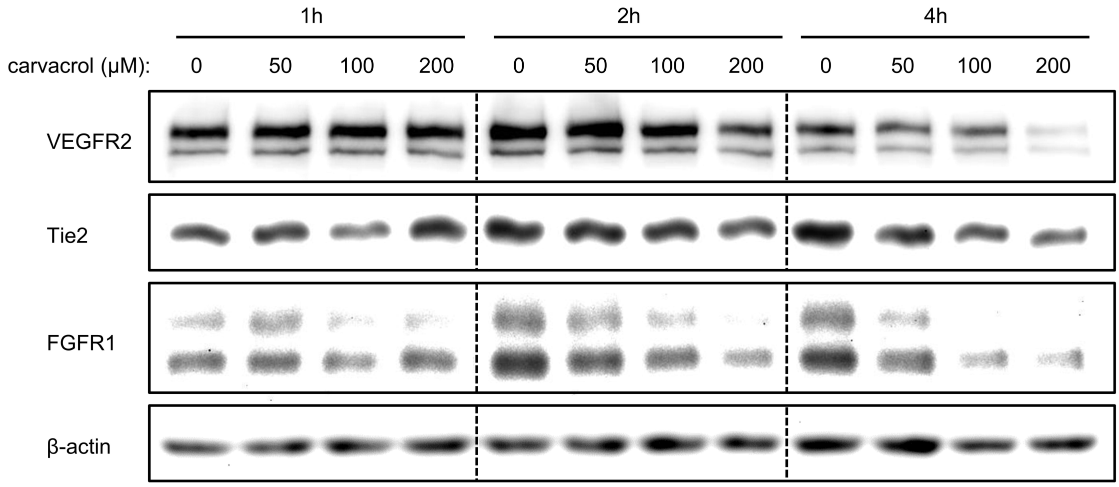

Supplementary Fig. 1 Effects of carvacrol on the protein levels of VEGFR2, Tie2, and FGFR1 in ECs after 1, 2, and 4 h of treatment.

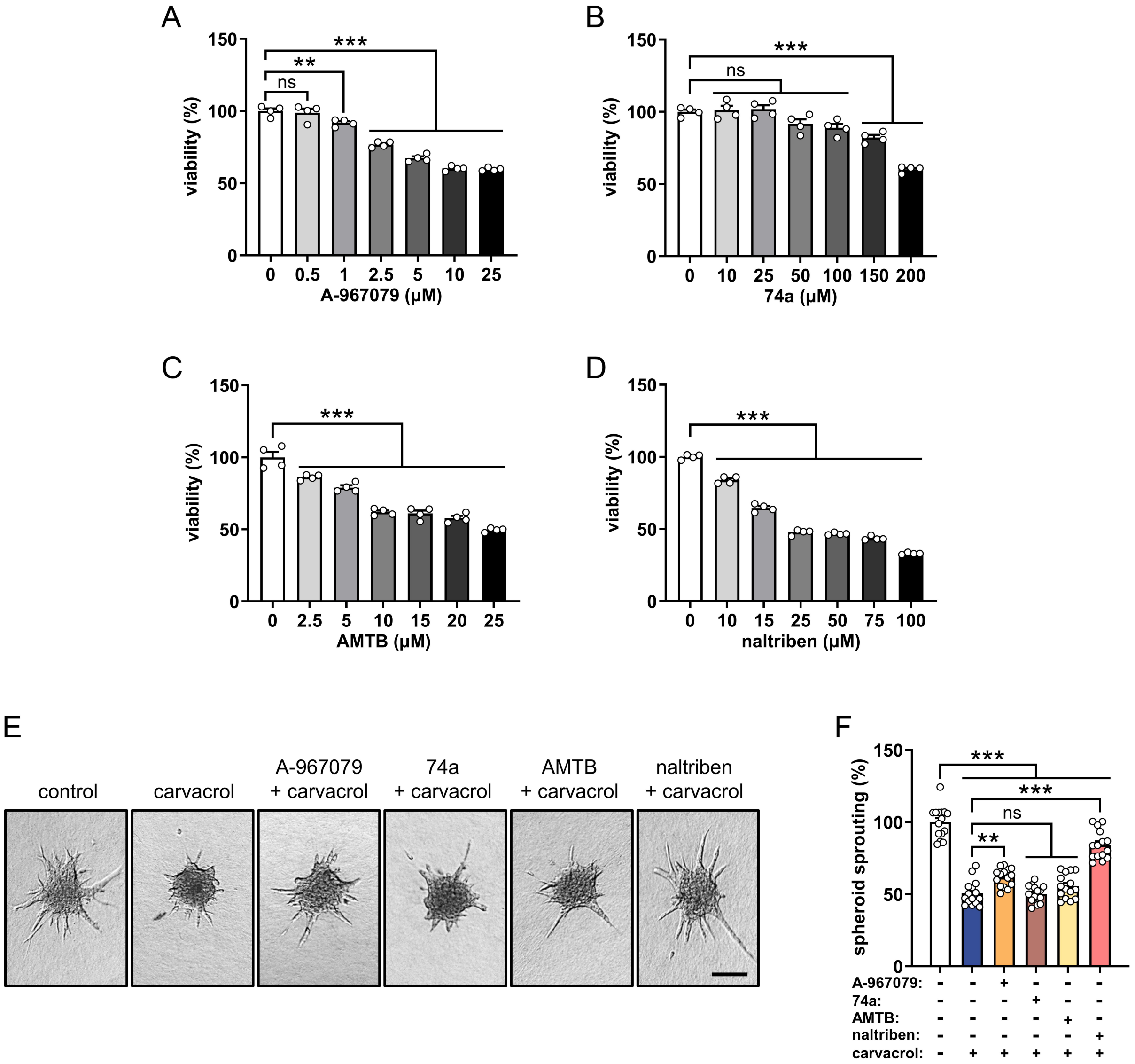

Supplementary Fig. 2 Carvacrol inhibits angiogenesis mainly through TRPM7 antagonism in ECs. **A-D:** Viability (% of 0 µM) of HUVECs that were treated for 24 h with a serial dilution of A-967079 (TRPA1 inhibitor; A), 74a (TRPV3 inhibitor; B), AMTB (TRPM8 inhibitor; C), or naltriben (TRPM7 channel activator; D), as assessed by WST-1 assay (n = 4). **E:** Representative images of HUVEC spheroids that were treated for 24 h with 0.1% DMSO (vehicle) or 200 µM carvacrol in the absence or presence of 2.5 µM A-967079, 150 µM 74a, 5 µM AMTB, or 10 µM naltriben. Scale bar: 65 µm. **F:** Sprouting (% of control) of treated HUVEC spheroids depicted in (E), as assessed by spheroid sprouting assay (n = 15). Data are presented as means ± SEM. $^{**}P < 0.01$, $^{***}P < 0.001$; ns, not significant.

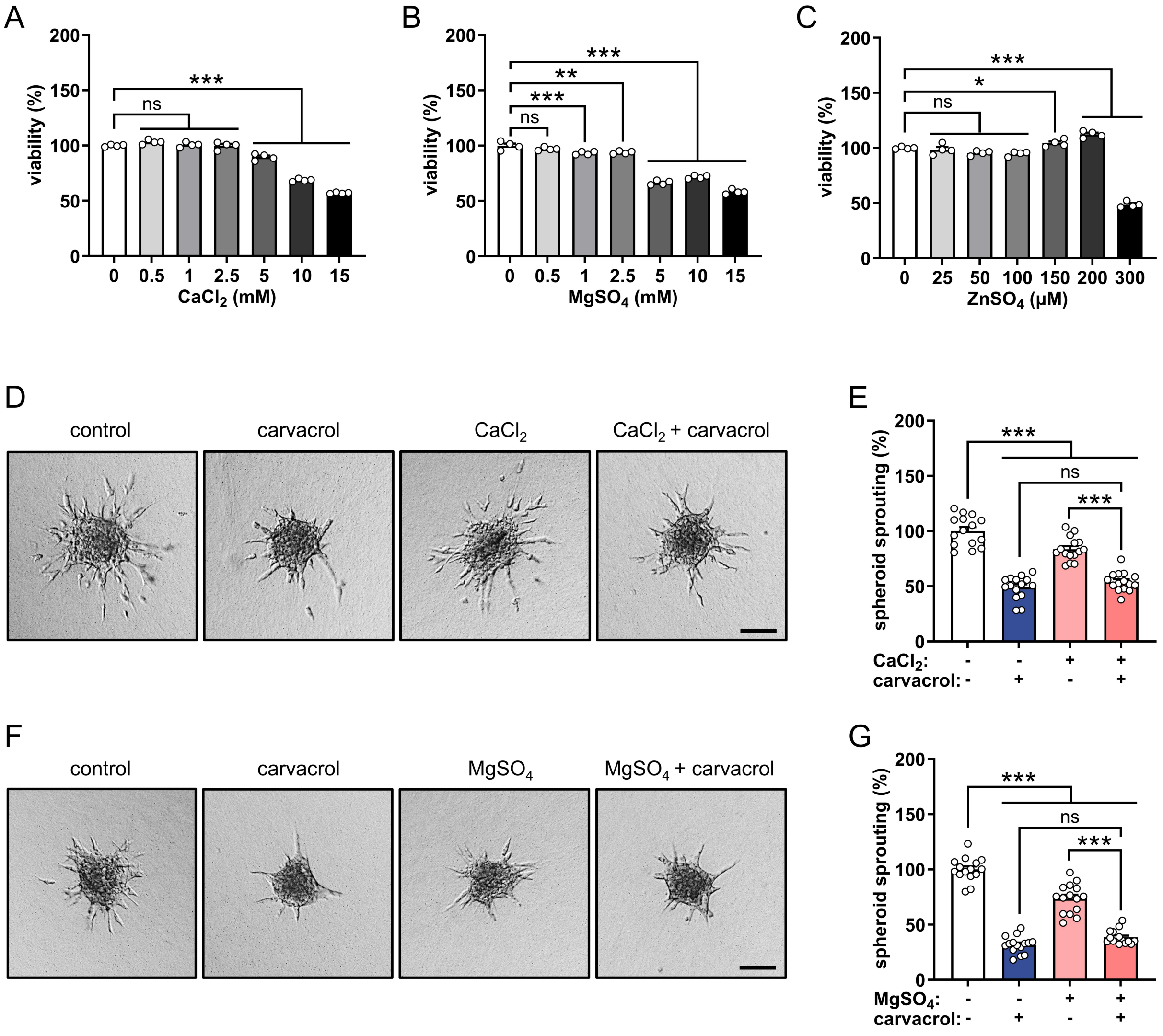

Supplementary Fig. 3 Carvacrol inhibits angiogenesis independently of TRPM7-mediated Ca^{2+} or Mg^{2+} influx in ECs. **A-C:** Viability (% of 0 mM or µM) of HUVECs that were treated for 24 h with a serial dilution of $CaCl_2$ (A), $MgSO_4$ (B), or $ZnSO_4$ (C), as assessed by WST-1 assay (n = 4). **D:** Representative images of HUVEC spheroids that were treated for 24 h with 0.1% DMSO (vehicle) or 200 µM carvacrol in the absence or presence of 5 mM $CaCl_2$. Scale bar: 60 µm. **E:** Sprouting (% of control) of treated HUVEC spheroids depicted in (D), as assessed by spheroid sprouting assay (n = 15). **F:** Representative images of HUVEC spheroids that were treated for 24 h with 0.1% DMSO (vehicle) or 200 µM carvacrol in the absence or presence of 2.5 mM $MgSO_4$. Scale bar: 60 µm. **G:** Sprouting (% of control) of treated HUVEC spheroids depicted in (F), as assessed by spheroid sprouting assay (n = 15). Data are presented as means ± SEM. *$P < 0.05$, **$P < 0.01$, ***$P < 0.001$; ns, not significant.

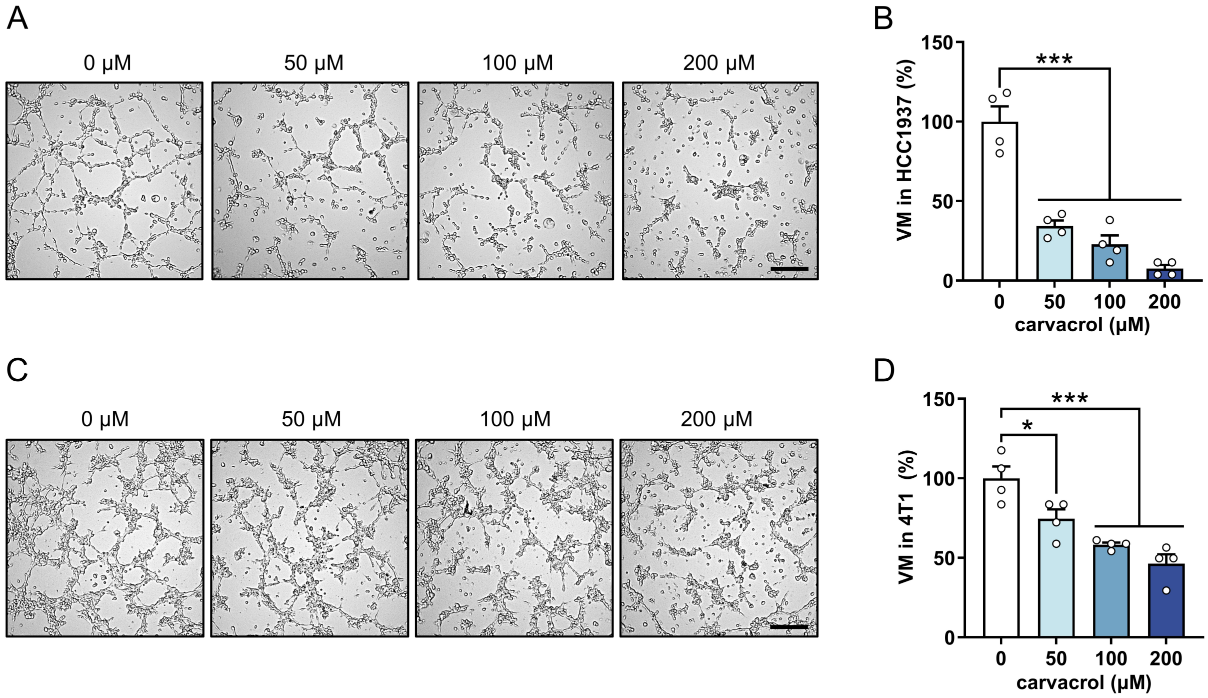

Supplementary Fig. 4 Carvacrol inhibits VM in HCC1937 and 4T1 cells. **A:** Representative images of tube-forming HCC1937 cells that were treated for 18 h with 0, 50, 100, and 200 μM carvacrol. Scale bar: 200 μm. **B:** VM (% of 0 μM) in treated HCC1937 cells depicted in (A), as assessed by tube formation assay (n = 4). **C:** Representative images of tube-forming 4T1 cells that were treated for 18 h with 0, 50, 100, and 200 μM carvacrol. Scale bar: 200 μm. **D:** VM (% of 0 μM) in treated 4T1 cells depicted in (C), as assessed by tube formation assay (n = 4). Data are presented as means ± SEM. *$P < 0.05$, ***$P < 0.001$.

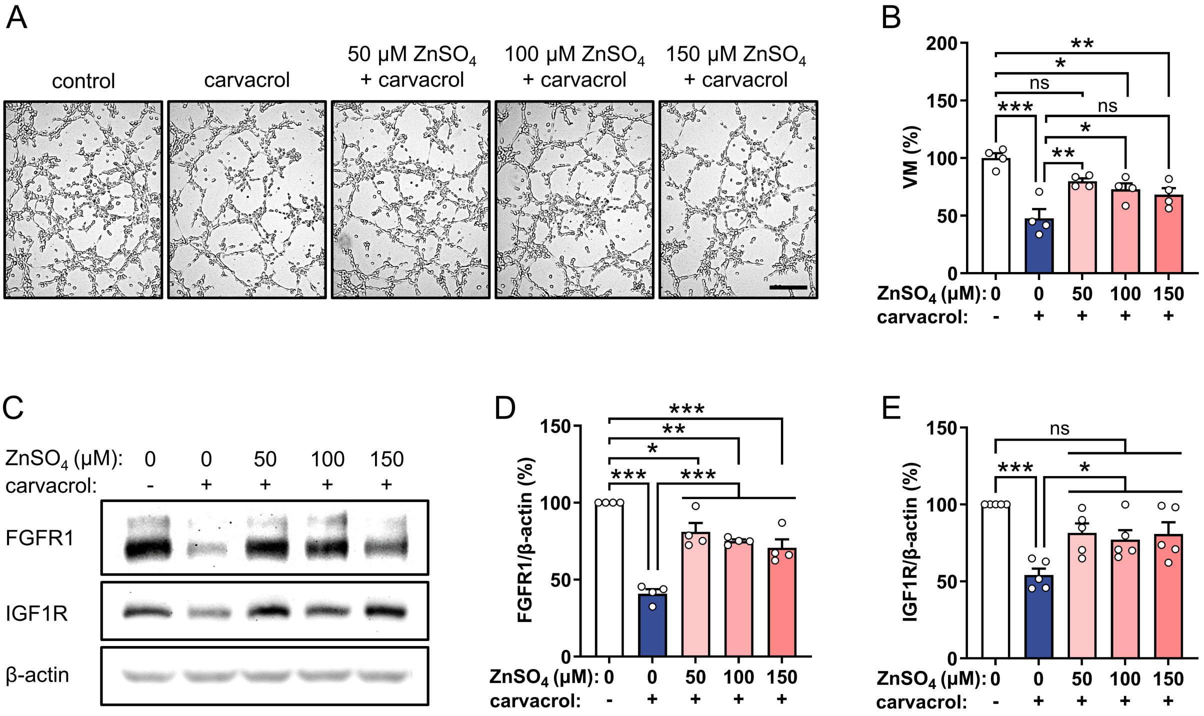

Supplementary Fig. 5 Carvacrol inhibits VM through blocking TRPM7-mediated Zn^{2+} influx in TNBC cells. **A:** Representative images of tube-forming MDA-MB-231 cells that were treated for 18 h with 0.1% DMSO (vehicle) or 50 μM carvacrol in the presence of 0, 50, 100, or 150 μM $ZnSO_4$. Scale bar: 200 μm. **B:** VM (% of control) of treated MDA-MB-231 cells depicted in (A), as assessed by tube formation assay (n = 4). **C:** Representative Western blots showing FGFR1, IGF1R, and β-actin expression in MDA-MB-231 cells that were pre-treated with 0, 50, 100, or 150 μM $ZnSO_4$ for 2 h and then exposed to 0.1% DMSO (vehicle) or 200 μM carvacrol for another 4 h. **D, E:** Expression levels (% of control) of FGFR1 (D) and IGF1R (E) normalized to β-actin in treated MDA-MB-231 cells depicted in (C), as assessed by Western blotting (n = 4-5 independent experiments). Data are presented as means ± SEM. $*P < 0.05$, $**P < 0.01$, $***P < 0.001$; ns, not significant.

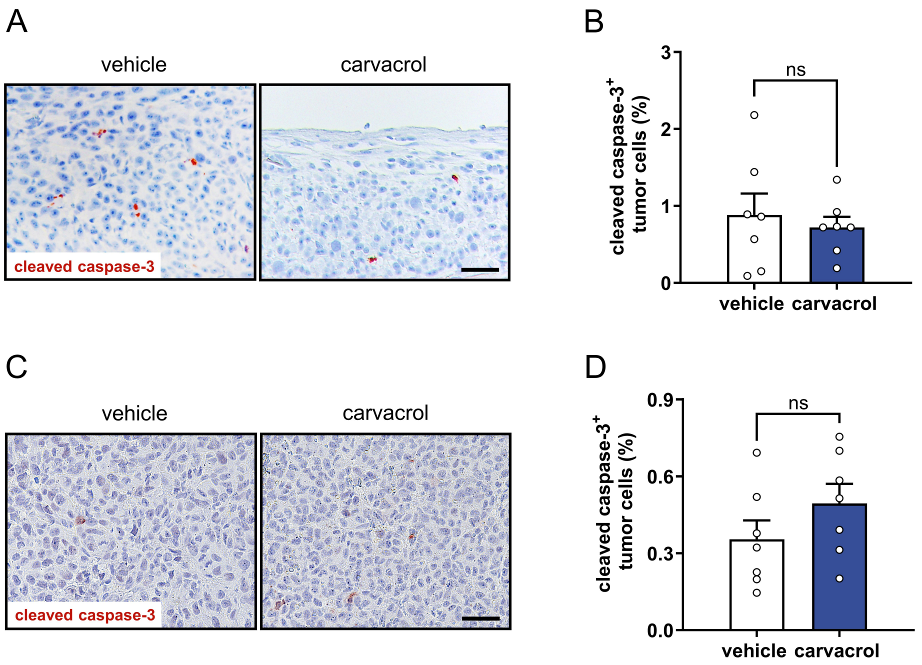

Supplementary Fig. 6 Carvacrol shows no effects on cell apoptosis of 4T1 tumors and MDA-MB-231 tumors. **A:** Representative images of cleaved caspase-3-stained sections of 4T1 tumors from vehicle- and carvacrol-treated mice on day 14 after spheroid transplantation. Scale bars: 40 µm. **B:** Cleaved caspase-3$^+$ tumor cells (% of total cell number) in 4T1 tumors depicted in (A), as assessed by immunohistochemical staining of cleaved caspase-3 (n = 7). **C:** Representative images of cleaved caspase-3-stained sections of MDA-MB-231 tumors from vehicle- and carvacrol-treated mice on day 42 after tumor inoculation. Scale bars: 40 µm. **D:** Cleaved caspase-3$^+$ tumor cells (% of total cell number) in MDA-MB-231 tumors depicted in (C), as assessed by immunohistochemical staining of cleaved caspase-3 (n = 7). Data are presented as means ± SEM. ns, not significant.
